# The effectiveness of a theory-based intervention program for pregnant women with anemia: A randomized control trial

**DOI:** 10.1371/journal.pone.0278192

**Published:** 2022-12-06

**Authors:** Raudah Abd Rahman, Idayu Badilla Idris, Zaleha Md Isa, Rahana Abd Rahman

**Affiliations:** 1 Public Health Division, Kuala Lumpur and Putrajaya Health Department, Kuala Lumpur, Wilayah Persekutuan, Malaysia; 2 Department of Community Health, Faculty of Medicine, Universiti Kebangsaan Malaysia Jalan Yaacob Latif, Cheras, Kuala Lumpur, Malaysia; 3 Department of Obstetrics and Gynaecology, Faculty of Medicine, Universiti Kebangsaan Malaysia, Kuala Lumpur, Malaysia; PLOS: Public Library of Science, UNITED KINGDOM

## Abstract

Anemia in pregnancy is a public health concern. It has been diagnosed in 27% of pregnant women in Malaysia and up to 40% of pregnant women globally. This study aimed to develop and evaluate the effectiveness of an intervention initiative based on the health belief model. The MyPinkMom program was disseminated through a mobile messaging application to pregnant women to educate them on the prevention of anemia in pregnancy. We conducted a two-arm cluster-assignment, single-blinded, randomized control trial at two government antenatal clinics in Selangor. One clinic was randomly chosen as the intervention group, and the other was chosen as the control group. Sixty pregnant women with anemia from the intervention group received the MyPinkMom intervention program in the form of six infographic video clips, and 60 pregnant women with anemia from the control group received routine counseling on anemia in pregnancy. Pregnant women who had anemia secondary to hemoglobinopathy or other chronic diseases were excluded from this study. MANOVA showed significant increases in hemoglobin, knowledge, attitude, subjective norms, and perceived behavioral control scores for adherence to iron supplements, dietary iron, and dietary vitamin C intake (p < 0.001) in the intervention group at week 6. A significant reduction also occurred in dietary tannin intake (p < 0.001) in the intervention group at week 6. The intervention group at week 6 showed a large effect on hemoglobin level increments (partial eta squared, Ƞp2 0.268), dietary iron intake (Ƞp2 0.213), knowledge of anemia in pregnancy (Ƞp2 0.622), subjective norm scores for adherence to iron supplements (Ƞp2 0.167), and reduction in dietary tannin intake (Ƞp2 0.353). Similarly, repeated measures ANOVA showed that changes in hemoglobin levels were significantly different over time (i.e., at baseline, week 6, and week 12) between the intervention and control groups (p < 0.001). Hemoglobin increased rapidly over time among participants in the intervention group but gradually in the control group. To conclude, the newly developed MyPinkMom program that was delivered through a messaging application showed effectiveness in preventing anemia during pregnancy.

## Introduction

Anemia has been a significant public health problem worldwide, especially in developing countries, including Malaysia. The World Health Organization has defined anemia in pregnancy as hemoglobin of less than 11 g/dL [1]. The commonest cause of anemia in pregnancy is iron deficiency anemia, defined as serum ferritin of less than 15μg/L [2]. Hereditary anemia such as thalassemia and hemoglobin E is also prevalent, especially in the South-East Asian region, and may also cause anemia among pregnant women [3]. The estimated prevalence of α-thalassemia and hemoglobin E or β-thalassemia in Malaysia is 17% and 34%, respectively [4, 5]. In addition, infections such as malaria, human immunodeficiency virus (HIV), and tuberculosis, and chronic diseases such as chronic renal disease and some types of cancer, may cause anemia among pregnant women [6]. These different types of anemia including inherited anemia and chronic diseases may result in poor maternal and perinatal outcomes. Therefore, pregnant women with anemia require specialist referral, as well as following routine antenatal management [7].

The prevalence of anemia in pregnancy is 40% worldwide [8]. In Malaysia, the prevalence of anemia in pregnancy was 27% in 2011 [8] and increased to 29.3% in 2016 [9]. Despite a routine nationwide prophylactic supplement program in Malaysia, the prevalence of anemia in pregnancy in several states has been reported to be high e.g., 33.0% in urban Selangor [10], 43.6% in urban Perak [11], and as high as 57.4% in Jerteh, Terengganu [12].

The risk of maternal death is two times higher with anemia in pregnancy [13]. This could be secondary to severe hemorrhage during delivery [14] and cardiac failure [14, 15] or both, which can be associated with severe anemia. Examples of perinatal morbidity are small size for gestational age [14, 16], low birth weight [17], prematurity [16, 17], and the risk of anemia among newborns [18, 19].

Many clinical trials have proven the effectiveness of iron supplementation in preventing iron deficiency anemia among pregnant women [20–24]. However, in reality, iron supplementation often did not prevent maternal anemia because most pregnant women with anemia were non-compliant with iron tablet regimes that were prescribed according to routine guidelines [25–27]. This was also influenced by several factors including poor knowledge of anemia [28, 29] and mothers’ unfavorable attitudes to adhering to daily iron tablet intake, such as being forgetful or fear of side effects [25, 27, 30].

Correspondingly, educational intervention programs using traditional methods of promoting health education have been proven to improve knowledge about the hemoglobin levels of pregnant women with anemia [31–33]. However, with the existence of smartphones and available network coverage worldwide, the delivery of healthcare education, services, and health promotion through mobile telecommunication technology is now more feasible [34]. More than five billion wireless subscribers exist worldwide, 70% of them from low- to middle-income countries [34]. Nevertheless, educational interventions using mobile telecommunication technology regarding anemia in pregnancy are still uncommon, especially in this region. Generally, the current approach to educating pregnant women on anemia in antenatal clinics in Malaysia is during dietary counseling by nutritionists and staff nurses, and this requires more time and human resources. Therefore, the use of mobile telecommunication technology in educating pregnant women with anemia can be an alternative approach to improve pregnant mothers’ hemoglobin levels and prevent subsequent maternal and perinatal poor outcomes.

Some intervention studies on anemia in pregnancy using mobile telecommunication technology have been conducted in other countries, such as in India by Pai et al. (2013) [35] and in Iran by Khorshid et al. (2014) [36]. Pai et al. (2013) [35] used automated voice calls for reminders on iron supplement consumption during an intervention study for anemia in pregnancy in India. Khorshid et al. (2014) [36] used text-messaging information on iron supplementation for pregnant women with anemia in Iran. However, this method of delivery is still lacking in Malaysia.

The general objective of this study was to assess the effectiveness of a newly developed and validated intervention program called MyPinkMom, using a randomized control trial. The MyPinkMom program was developed based on the health belief model and disseminated to pregnant women using mobile technology to educate them on the prevention of anemia in pregnancy. The primary objective was to compare the hemoglobin levels of the pregnant women in the intervention and control groups. The secondary objectives were to compare the pregnant women’s knowledge of anemia in pregnancy, their attitudes about adherence to daily iron tablets throughout pregnancy, and dietary iron intake among participants from the intervention and control groups.

## Materials and methods

### Development and validation of MyPinkMom intervention program

We developed the MyPinkMom intervention program using the Analysis, Design, Development, Implementation, and Evaluation (ADDIE) model, a systematic structural design model for designing educational programs [37, 38]. The development was conducted in five phases: analysis, design, development, implementation, and evaluation.

During the ***analysis phase***, we studied all related clinical guidelines, literature reviews, and existing educational materials for pregnant women with anemia to ensure that content from these evidence-based materials was included in the intervention program. The content was discussed among experts consisting of public health physicians, obstetricians, and nutritionists. Subsequently, during the ***design phase***, the selected materials were converted into interesting educational materials, based on experts’ discussions. Altogether, there were six sets of education materials, each with its own predetermined objectives. The content of each set or session was structured based on the health belief model construct, i.e., perceived susceptibility, perceived severity, perceived benefit, and perceived barrier, and their respective objectives, which are shown in Table 1.

**Table 1:** Health belief model constructs, objectives, and outlines of content for the MyPinkMom program.

MyPinkMom program	Health belief model construct	Objective	Outline of content
Video 1: Definition of hemoglobin and anemia in pregnancy	Perceived susceptibility	To explain general information on hemoglobin and anemia in pregnancy	• Explanation of hemoglobin and its function • Definition of anemia in pregnancy • Common causes of anemia in pregnancy
Video 2: Complications of anemia in pregnancy	Perceived severity	To explain the complications of anemia in pregnancy	• Description of complications of anemia in pregnancy for both mothers and babies
Video 3: Pathophysiology of anemia in pregnancy	Perceived susceptibility	To explain why pregnant women can easily develop anemia and how to detect anemia	• Information on the pathophysiology process that explains why pregnant women are at higher risk for developing anemia • Education on how to recognize signs and symptoms of anemia
Video 4: Proper dietary intake for pregnant women with anemia	Perceived benefit	To explain proper dietary intake to prevent and correct anemia during pregnancy	• Description of iron-rich, iron-enhancer, and iron-inhibitor foods in the diet
Video 5: Proper iron supplement intake for pregnant women with anemia	Perceived benefit	To educate participants on proper iron supplement intake	• Education on the availability of different types of iron supplements and proper iron supplement intake to prevent anemia
Video 6: Possible side effects of oral iron tablets and how to handle them	Perceived barrier	To educate participants on possible side effects of oral iron tablets and ways to handle them	• Education on the possible side effects of iron tablets and steps to handle them

During the ***development phase***, the MyPinkMom program was then developed into PowerPoint presentation slides based on the objectives, the content outline, and literature reviews. The PowerPoint slides were subsequently converted into infographic video clips with audio explanations and interesting animations, arranged by a computer graphic designer.

In the following ***implementation phase***, MyPinkMom infographic video clips were shown to five experts again (two public health physicians, an obstetrician, and two nutritionists), as well as 10 pregnant women, to evaluate the appropriateness, usefulness, and comprehensibility of the content and the presentation of the infographic videos. Based on the findings from the evaluation phase, corrections were made before they were used in the actual intervention study.

In the ***evaluation phase***, each expert rated each video as 1 for “very irrelevant,” 2 for “irrelevant,” 3 for “relevant,” or 4 for “very relevant.” Following this, the Item-Content Validity Index (I-CVI) was calculated. This was the number of experts in mutual agreement and who gave total ratings of 3 or 4 divided by the total number of experts involved for each video clip. The Scale-Content Validity Index (S-CVI), which was the average of the I-CVI, was also calculated. The I-CVI value for each video clip ranged from 0.8 to 1.0. The S-CVI value was 0.97. The acceptable value of S-CVI is 0.80 if the number of experts involved is five or fewer [39]. These findings conclude that both were acceptable.

### The intervention study

#### Study design

A two-arm cluster-assignment, single-blinded, randomized controlled trial (RCT) with a 1:1 allocation ratio was conducted in health clinics and groups with participants (pregnant women with anemia) in the Petaling district, which is one of the most populated districts in Selangor, Malaysia. Two government antenatal clinics were randomly selected as the study sites. One clinic was assigned as the intervention clinic, and the other clinic was selected as the control clinic. This was to avoid possible contamination of information between participants if randomization was to be conducted at the same clinic.

#### Study population

The study population was pregnant women who attended both antenatal clinics and had been diagnosed with anemia (i.e., hemoglobin less than 11 g/dL).

#### Sampling methods

We applied multistage random sampling during this RCT. In the earlier phase, the Petaling district was selected using purposive sampling. Subsequently, 60 pregnant women who had been diagnosed with anemia and fulfilled the eligibility criteria from one clinic that was chosen randomly as the intervention site were randomly selected to be the intervention group. Another 60 pregnant women with anemia who fulfilled the eligibility criteria from another clinic that was randomly selected from the control clinic were randomly selected to be the control group. The randomization was conducted using a random number generator.

#### Inclusion criteria

The participants were enrolled from mid-November until mid-December 2019. Pregnant women between 13 and 24 gestational weeks, aged between 20 and 40 years, with a hemoglobin level at recruitment of 7.0 to 10.9 g/dL, who could read and write in Malay or English, and had access to a smartphone with WhatsApp were included in this study by researcher. In cases where pregnant women fulfilled all eligibility criteria except owning a smartphone or having internet access, MyPinkMom videos were shown to them using the researcher’s smartphone during their follow-ups. None of the participants were excluded for that reason.

#### Exclusion criteria

Pregnant women were excluded if they had underlying diseases such as hemoglobinopathy (thalassemia, hemoglobin E), chronic diseases (chronic renal disease, cancer), or infectious diseases (HIV, malaria), presented with symptoms of anemia (shortness of breath, chest pain, syncope) during this current pregnancy, or planned for termination of the pregnancy. These patients were referred for appropriate treatment according to their respective diseases.

#### Implementation of the intervention program

In an earlier study protocol, we planned to deliver the intervention in 2 weeks, where three videos would be delivered per week followed by the evaluation at weeks 4 and 8. However, some changes were made. The intervention was carried out for the whole 6 weeks. During week 1, each video was disseminated daily for 6 days, followed by a weekly reminder in weeks 2 to 5. These changes were made because of logistic problems and practicality.

We used WhatsApp to deliver the videos to the participants in the intervention group. Each video session lasted for approximately 3 to 5 minutes. In the control group, participants received treatment as usual: an explanation of anemia in pregnancy by the nurses who applied the information available in the antenatal books.

Both the intervention and control groups received routine antenatal care including folate and iron tablet supplements such as Zincofer, Maltofer, Ferrous, Iberet, or Obimin. Participants from both the intervention and control groups were prohibited from taking medications or vitamins other than those prescribed by the doctors.

#### Study outcomes

The primary outcome was hemoglobin level, and the secondary outcomes were knowledge scores on anemia in pregnancy, attitude towards adherence to iron supplements using the theory of planned behavior construct (i.e., attitude, subjective norms, perceived behavior control), and the intake of dietary iron, iron enhancers, (vitamin C) and iron inhibitors (phytate, tannin, calcium).

#### Participant timeline

The total duration of participants’ involvement in this research was 12 weeks. The intervention was carried out from week 1 to week 5. The baseline assessment was carried out at week 1 before the intervention commenced. The post-intervention test was carried out at week 6 (immediately post intervention) and again at week 12 (6 weeks post intervention).

#### Sample size calculation

The sample size was calculated using the following formula for a clinical trial comparing the means of two groups [39].

n = [(Zα/2+Zβ)^2^ × (ó1 + ó2)^2^)]/ (μ1 –μ2)^2^, where

n = sample size per arm

μ1 = mean hemoglobin of the intervention group after the intervention in a previous study (11.97 g/dL) [40].

Μ2 = mean hemoglobin of the control group after the intervention in a previous study (11.1g/dL) [34]

Μ1-μ2 = difference of mean hemoglobin in the intervention and control groups after the intervention (11.97–11.1 = 0.87) [34]

Ó1 = standard deviation for the intervention group (0.9) [34]

Ó2 = standard deviation for the control group (1.3) [34]

Zα/2 = 1.96 with a significance level of 5%

Zβ = 0.84 with power of 80%.

Therefore, the sample size for this study when calculated was 50 per arm. The total sample size was 120, taking into account a 20% dropout rate.

#### Blinding

The participants were the only ones blinded in this study. They were unaware whether they belonged to the intervention or control group. However, the investigators and data analyzer were not blinded, and they were aware of the intervention received by the participants.

#### Data collection

**Fig 1:**
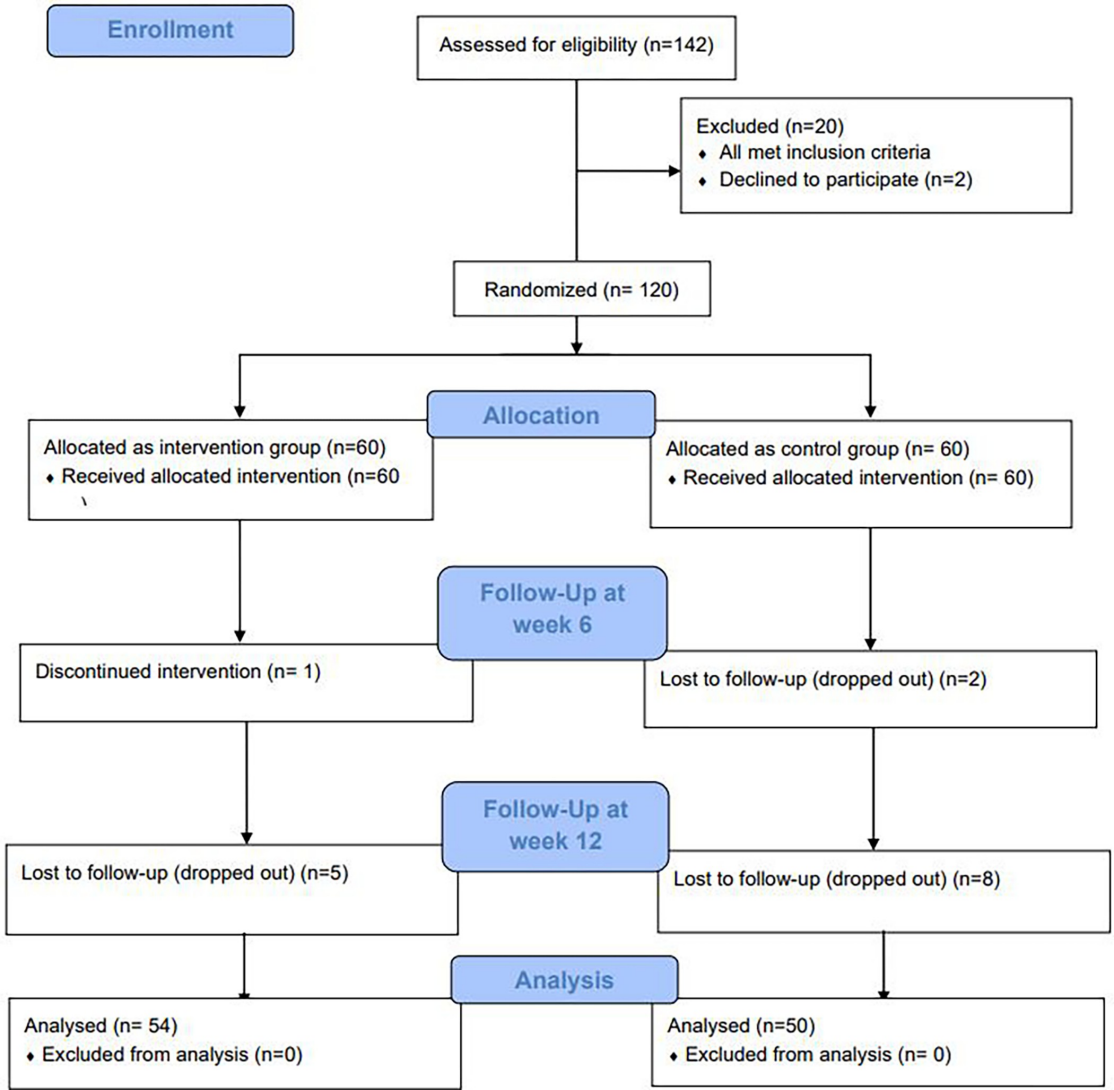
CONSORT participant flow diagram.

The baseline assessment that was conducted before the intervention included recording the hemoglobin level at recruitment, obtaining participants’ sociodemographic characteristics, obstetric profile, dietary iron, iron enhancer and iron inhibitor intake, knowledge scores on anemia in pregnancy, and attitude scores toward adherence to iron tablet intake. The post-intervention assessment used the same measures, excluding sociodemographic characteristics and obstetric profile; this was conducted at week 6 (immediately post intervention). In addition, the hemoglobin level was assessed again at week 12 (6 weeks post intervention). The consort diagram is ilustrated in Fig 1.

Hemoglobin was measured by performing a full blood count, which is normally tested for pregnant women with anemia in antenatal clinics for routine monitoring of hemoglobin. Investigations such as serum ferritin level, hemoglobin electrophoresis, HIV screening, and renal profile were performed as routine antenatal management in the respective clinics only when determining etiological causes of anemia. Hemoglobin electrophoresis for mothers was only carried out during antenatal follow-ups when there was no improvement in the hemoglobin level after mothers had complied with iron and folic acid tablet consumption.

Attitudes towards adherence to iron supplementation were measured using a theory of planned behavior–based questionnaire that measured adherence to iron supplements. Dietary iron, iron enhancer, and iron inhibitor intake were also measured using the validated Food Frequency Questionnaire (FFQ) for pregnant women [41].

#### Data management and data analysis plan

Data were analyzed using SPSS software. For descriptive analysis, categorical data were summarized descriptively as frequencies and percentages, and numerical data were summarized as means and standard deviations. All sociodemographic characteristics and outcome measurements at baseline for both participants from the intervention and control groups were compared using Student’s t-test for normally distributed continuous factors. Categorical data were compared using the chi-square test for sufficiently large sample sizes or Fisher’s exact test for smaller sample sizes.

All outcome measurements between the intervention and control groups at week 6 were compared using multivariate analysis of variance (MANOVA). Finally, the changes in hemoglobin over time (at baseline, week 6, and week 12) between the intervention and control groups were compared using repeated measures ANOVA. The level of significance was predetermined at p-values of less than 0.05.

#### Ethics consideration

Ethical approvals from the Universiti Kebangsaan Malaysia (UKM) Research and Ethics Committee (UKM PPI/111/8/JEP-2018-674) and the Medical Research Ethical Committee (MREC) of the Ministry of Health Malaysia through the National Medical Research Register (NMRR; NMRR-18-2612-43423) were obtained before the study was conducted. Approval was also obtained from the Selangor State Health Department authority.

This study was ethically conducted according to the Declaration of Helsinki. Participation was voluntary. The participants were given adequate time to read the patient information sheet and written informed consent. Each participant was briefly informed about the study by the researcher, and they were allowed to ask any questions. Patients who signed the consent form and agreed to participate in the study were included in this study.

Participants’ confidentiality was always preserved throughout the study and during its presentation and publication. No identifying data were revealed in the questionnaire or will be in the future. The participants were not given access to their personal information and study data. However, they can be informed about the research findings if they wish to know. After the intervention period was completed, MyPinkMom program videos were also delivered to the participants in the control group.

## Results

The sociodemographic characteristics of the participants are shown in Table 2. The majority were Malay (77.5%), nonworking or housewives (61.7%), and with lower education (75.8%). The mean age of participants was 28 years.

**Table 2:** Sociodemographic characteristics of participants.

	Frequency (%)	Mean (SD)
	n = 120	n = 120
Race:		
Malay	93 (77.5)	
Chinese	12 (10.0)	
Indian	8 (6.7)	
Others	7 (5.8)	
Age		28.49 (5.046)
Age group:		
<30 years old	80 (66.7)	
30–40 years old	40 (33.3)	
Education:		
Diploma and higher	29 (24.2)	
Secondary school and lower	91 (75.8)	
Occupation:		
Housewife	74 (61.7)	
Working (employed/self-employed)	46 (38.3)	
Gravida:		
Primigravida	53 (44.2)	
Non-primigravida	67 (55.8)	
Hemoglobin at booking		11.13 (0.953)
Type of iron supplement prescribed:		
Zincofer	79 (65.8)	
Iberet	24 (20.0)	
Obimin	17 (14.2)	

SD = standard deviation

Using Student’s t-test and the chi-square test, the characteristics of participants in the intervention and control groups were not significantly different (p > 0.05), as shown in Table 3.

**Table 3:** Comparison of participants’ characteristics in intervention and control groups at baseline.

	Intervention n (%) n = 60	Control n (%) n = 60	x^2^ value ^a^	*p*-value ^a^
Race:			0.048	0.827
Malay	46 (76.7%)	47 (78.3%)		
Non-Malay	14 (23.3%)	13 (21.7%)		
Age:			1.350	0.245
<30 years old	43 (71.7%)	37 (61.7%)		
30–40 years old	17 (28.3%)	23 (38.3%)		
Education:			2.228	0.136
Diploma and higher	18 (30.0%)	11 (18.3%)		
Secondary school and lower	42 (70.0%)	49 (81.7%)		
Occupation:			0.564	0.453
Housewife	35 (58.3%)	39 (65.0%)		
Working (employed/self-employed)	25 (41.7%)	21 (35.0%)		
Gravida:			2.737	0.098
Primigravida	31 (51.7%)	22 (36.7%)		
Multigravida	29 (48.3%)	38 (63.3%)		
Type of iron supplement:			3.753	0.153
Zincofer	34 (56.7%)	44 (73.3%)		
Iberet	15 (25.0%)	10 (16.7%)		
Obimin	11 (18.3%)	6 (10.0%)		
	Mean (SD)	Mean (SD)	t-value ^b^	p-value ^b^
	n = 60	n = 60		
Age, years	28.27 (4.411)	28.72 (5.639)	-0.487	0.627
Gestational week	22.45 (2.547)	21.53 (3.078)	4.400	0.078
Gravida	2.20 (1.527)	2.57 (1.845)	0.720	0.238
Hemoglobin, g/dL	10.16 (0.526)	10.01 (0.676)	1.387	0.168
Knowledge score	16.63 (4.647)	16.53 (4.945)	0.114	0.909
Attitude score	5.91 (0.911)	5.93 (0.927)	-0.132	0.895
Subjective norms score	6.07 (0.884)	5.75 (0.998)	1.815	0.072
Perceived behavioral control score	5.717 (1.493)	5.367 (1.362)	1.341	0.182
Dietary iron (mg/day)	15.39 (5.966)	15.41 (3.917)	-0.024	0.981
Dietary vitamin C (mg/day)	115.10 (116.055)	111.88 (116.247)	-0.152	0.880
Dietary tannin (mg/day)	468.58 (568.522)	592.83 (534.645)	-1.233	0.220
Dietary phytate (mg/day)	458.28 (274.485)	413.58 (273.820)	0.893	0.374
Dietary calcium (mg/day)	885.93 (759.991)	695.57 (616.920)	1.506	0.135

^a^Chi-square test

^b^Student’s t-test, p-value level at 0.05, SD = standard deviation

A baseline assessment was carried out for all 60 participants in each group. At week 6, one participant from the intervention group was excluded because they received parenteral iron during the intervention period. During the same week, two participants from the control group dropped out. Thus, at week 6, the number of participants from the intervention group was 59, and the number of participants from the control group was 58. At week 12, five out of 59 participants from the intervention group, and eight out of 58 participants from the control group, dropped out of the study. Thus, the final total numbers of participants from the intervention and control groups who took part in the final post-test assessment were 54 and 50, respectively.

Table 4 illustrates the analysis of the between-subject effect. MANOVA was performed after all assumptions were met. As shown in this table, Wilk’s lambda test was significant in this study (p <0.001), which indicated significant differences for the dependent variables (outcome measurement) between the intervention and control groups [36] (Table 4).

**Table 4:** Overall effect of the MyPinkMom intervention program on all dependent variables at week 6 (immediately post intervention).

Effect		Value	F^c^	p-value^c^	Partial eta squared
Intercept	Wilk’s lambda	0.002	6534.64	<0.001	0.998
Group	Wilk’s lambda	0.226	36.29	<0.001	0.774

^c^MANOVA, p-value level at 0.05

The multivariate partial eta squared (Ƞ_p_^2^) value was 0.774, which means that 77.4% of the multivariate variance of the dependent variables, i.e., the outcome measurements at week 6 (immediately post intervention), were associated with the independent variables due to the intervention received for both the intervention and control groups [42, 43] (Table 4). In other words, the overall effect size of the MyPinkMom intervention on all dependent variables at week 6 (immediately post-intervention) was approximately 77.4%.

As shown in Table 5, the mean difference of each outcome measurement at week 6 (immediately post intervention) between the intervention and control groups, and the effects of the intervention on each outcome measurement (Ƞp^2^), were next determined. As shown in this table, the mean differences in hemoglobin level, knowledge score on anemia in pregnancy, dietary iron intake, dietary vitamin C intake, dietary tannin intake, attitude score, subjective norms score, and perceived behavior control score on adherence to iron supplementation immediately post intervention (at week 6) between the intervention and control groups were significantly different (*p* <0.05).

**Table 5:** Comparison of effect size and mean difference of each dependent variable between intervention and control groups at week 6 (immediately after the intervention).

Dependent variable/ outcome measurement	Group	n	Mean (SD)	Mean difference (95% CI)	p-value^d^	Ƞ_p_^2^
Hemoglobin (g/dL)	Intervention	59	10.75 (0.48)	0.66 (0.46, 0.87)	<0.001	0.27
	Control	58	10.08 (0.62)			
Dietary iron intake (mg/day)	Intervention	59	20.55 (5.12)	4.57 (2.95, 6.19)	<0.001	0.21
	Control	58	15.98 (3.60)			
Dietary vitamin C (mg/day)	Intervention	59	244.31 (233.87)	123.56 (54.53, 192.59)	0.001	0.10
	Control	58	120.76 (126.52)			
Dietary tannin (mg/day)	Intervention	59	86.94 (153.87)	-599.16 (-748.93, -449.39)	<0.001	0.35
	Control	58	686.10 (559.69)			
Dietary phytate (mg/day)	Intervention	59	377.63 (248.63)	-58.18 (-154.72, 38.35)	0.23	0.01
	Control	58	435.81 (277.39)			
Dietary calcium (mg/day)	Intervention	59	734.24 (611.712)	39.43 (-188.51, 267.37)	0.732	0.001
	Control	58	694.81 (632.584)			
Knowledge score	Intervention	59	26.41 (2.17)	7.30 (6.24, 8.36)	<0.001	0.62
	Control	58	19.10 (3.44)			
Attitude score	Intervention	59	6.44 (0.69)	0.56 (0.15, 0.26)	<0.001	0.11
	Control	58	5.88 (0.92)			
Subjective norm score	Intervention	59	6.46 (0.66)	0.90 (0.53, 1.27)	<0.001	0.17
	Control	58	5.56 (1.28)			
Perceived behavior control score	Intervention	59	6.31 (0.94)	0.80 (0.39, 1.20)	<0.001	0.12
	Control	58	5.51 (1.24)			

^d^MANOVA, *p*-value level at 0.05

The hemoglobin level, knowledge score on anemia in pregnancy, dietary iron intake, dietary vitamin C intake, attitude score, subjective norms score, and perceived behavior control score on adherence to iron supplementation at week 6 (immediately post-intervention) significantly increased in the intervention group when compared to the control group. The dietary tannin intake at week 6 (immediately post intervention) significantly decreased in the intervention group compared to the control group. However, the dietary phytate intake and dietary calcium intake at week 6 (immediately post intervention) were not significantly different between the intervention and control groups.

The effect size of the intervention on each dependent variable (outcome measurements) is determined by the Ƞp^2^ value, which measures the magnitude of the intervention’s effect on the participants [37]. It can be classified into small (Ƞp^2^≥0.01), medium (Ƞp^2^≥0.06), and large (Ƞp^2^≥0.14) effects [37]. The MyPinkMom program showed a large effect on the hemoglobin level increment, dietary iron intake, knowledge of anemia in pregnancy, subjective norm score on adherence to iron supplementation, and reduction in dietary tannin intake. This intervention had a medium effect on dietary vitamin C intake, attitude score, and perceived behavior control score on adherence to iron supplementation and only a small effect on dietary phytate intake. However, the MyPinkMom program did not affect dietary calcium intake.

Repeated measures ANOVA was conducted to evaluate the changes in hemoglobin level over time at baseline, at week 6 (immediately post intervention), and at week 12 (6 weeks post intervention). As observed in Table 6, changes in hemoglobin levels were significantly different over time between the intervention and control groups (F (1.724, 175.845) = 39.386, *p*<0.001).

**Table 6:** Changes in hemoglobin over time between intervention and control group.

Time	Intervention group, n = 54 Hemoglobin level (g/dL) Mean (SD)	Control group, n = 50 Hemoglobin level (g/dL) Mean (SD)	F stat (df) ^e^	p-value ^e^
Baseline assessment	10.20 (0.515)	10.12 (0.658)	39.386 (1.724;175.845)	<0.001
Week 6 (immediately post intervention)	10.79 (0.475)	10.21 (0.555)		
Week 12 (6 weeks post intervention)	11.48 (0.525)	10.41 (0.441)		

^e^ Repeated measures ANOVA, p*-*value level is at 0.05

**Fig 2:**
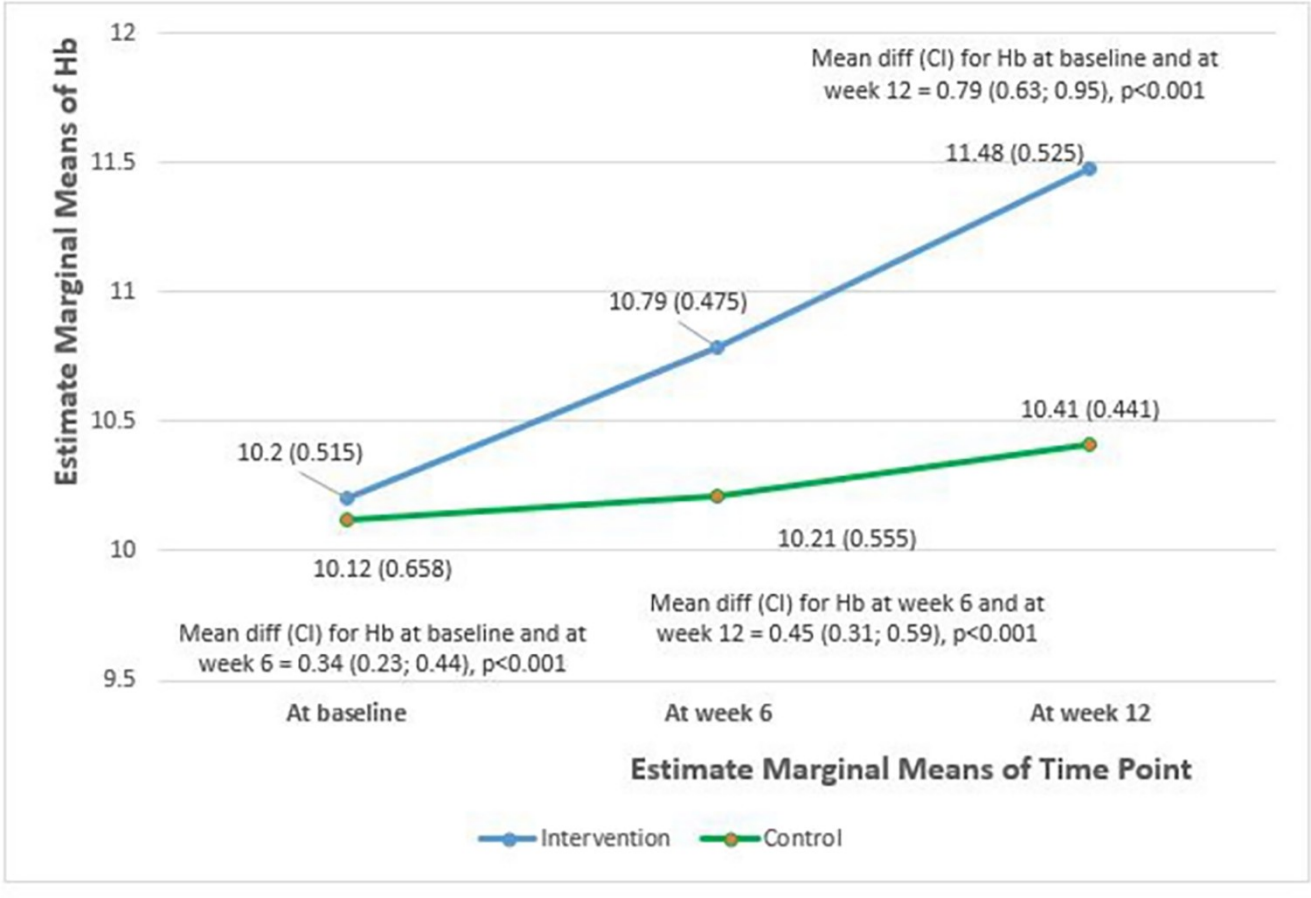
Changes of haemoglobin (Hb) over times between groups at baseline, week 6 and week 12 for intervention and control group.

Fig 2, which demonstrates the changes of hemoglobin over time at baseline, week 6, and week 12, shows an increment in hemoglobin level over time (at baseline, week 6, and week 12) for both groups. However, the hemoglobin level in the intervention group increased more rapidly over time (blue line), whereas the hemoglobin level of participants in the control group increased gradually over time (green line). The mean difference of hemoglobin at each time level (at baseline and week 6, at week 6 and week 12, and at baseline and week 12) was statistically significant. This shows a significant difference in hemoglobin changes at each time level.

## Discussion

The MyPinkMom program is a newly developed theory-based intervention program that was developed through evidenced and theoretical concepts. It may be the first intervention using mobile telecommunication technology for promoting health education to pregnant women with anemia in Malaysia. This intervention showed large effect sizes on all study outcomes including significant improvement in hemoglobin level, improvement in knowledge of anemia in pregnancy, improvement in dietary iron intake, and behavior towards adherence to iron supplementation. The changes in hemoglobin levels over time in the intervention group were more rapid compared to changes in the control group.

Other intervention studies have also used mobile technology. Similar to a study conducted in India, there was an increase in the hemoglobin level in the intervention group post intervention, but the increment was not statistically significant, and the effect size on the hemoglobin level was small [35]. In a study that used mobile text-messaging information on iron supplementation for pregnant women in Iran, this intervention also improved participants’ adherence to iron supplementation, but the increment of hemoglobin levels in the intervention group was not statistically significant [36]. However, unlike this present study, neither intervention study assessed the changes in hemoglobin levels over time [35, 36].

The positive outcome in this study may be explained by several strengths. Firstly, the MyPinkMom program was validated by experts. Secondly, it was developed based on theoretical concepts and extensive literature searches, including obtaining experts’ input. The content was comprehensive because it considered food or beverages to be taken together with iron supplements such as vitamin C–rich fruits, as well as food to be avoided such as coffee, tea, milk, and rice while consuming iron tablets or iron-rich food due to promotion or inhibition of iron absorption. The MyPinkMom program is based on the health belief model that promotes behavioral change, such as compliance with iron supplementation and dietary iron intake. The model emphasizes its constructs such as perceived susceptibility and perceived severity of anemia, as well as perceived benefits and perceived barriers to compliance with iron supplements and dietary iron intake throughout pregnancy.

Thirdly, this study applied mobile telecommunication technology as a means of delivering the intervention. Conveying educational intervention using mobile technology may be rare despite the increasing use of mobile smartphones. The content of this intervention was presented in interesting infographics with animations and clear audio explanations that provide better comprehensibility as opposed to the traditional way of health promotion.

Finally, the sample size for the intervention study was adequate. We used an RCT where 60 participants each were selected after eligibility screening from the intervention and control clinics after randomization. The randomization may minimize selection bias [44]. Participants were also blinded so that they were unaware of whether they belonged to the intervention or control group. This blinding can avoid the Hawthorne effect, which refers to behavioral change by participants when they are aware that they are being observed [45].

The study has some limitations. The study sample was pregnant women who attended two different antenatal clinics from two different areas in Petaling district, which is an urban area in Selangor. Thus, it does not represent the whole population in Malaysia, especially those from rural areas. The types of iron and folate supplements varied because they were prescribed by different doctors at both study sites. This may introduce a minimal effect of measurement bias in this study.

The possibility also existed of recall bias and measurement bias when answering the FFQ [46]. However, the recall bias was minimized by giving adequate time to the participants to answer the FFQs. The measurement bias was reduced by providing pictures of food according to serving sizes, which served as a guideline for the participants in answering the FFQs [47]. Biochemical marker measurements, such as serum ferritin and serum TIBC, have been found to be more accurate than dietary iron measurement because they are not associated with recall bias and measurement bias since these markers do not depend on the respondents’ ability to memorize their food intake [48]. However, these biochemical markers were not measured in this study because of financial constraints.

## Conclusions

This study has demonstrated that the newly developed MyPinkMom program that used mobile telecommunication technology is more effective than routine counseling in improving hemoglobin level, knowledge, dietary iron intake, and behavior towards adherence to iron supplementation among pregnant women with anemia. It can be considered as part of the management of pregnant women with anemia in this country.

## Supporting information

S1 Checklist CONSORT 2010 checklist of information to include when reporting a randomised trial*. (DOC)

S1 File (DOCX)
