# Supplementary material for: The effectiveness of a theory-based intervention program for pregnant women with anemia: A randomized control trial
Source: PLoS One. 2022 Dec 6;17(12):e0278192. doi: 10.1371/journal.pone.0278192 (PMC9725169; doi:10.1371/journal.pone.0278192)
Supplement: S1 File — (DOCX) [file pone.0278192.s002.docx]

RESEARCH TITLE:

DEVELOPMENT AND EFFECTIVENESS OF MYPINKMOM EDUCATIONAL MODULE FOR PREGNANT WOMEN WITH ANAEMIA IN PETALING DISTRICT

INVESTIGATORS:

RAUDAH BINTI ABD RAHMAN

IDAYU BADILLA BINTI IDRIS

ZALEHA BINTI MD ISA

RAHANA BINTI ABDUL RAHMAN

COMMUNITY HEALTH DEPARTMENT

FACULTY OF MEDICINE

UNIVERSITI KEBANGSAAN MALAYSIA

NMRR ID: NMRR-18-2612-4342

DATE: 5 NOVEMBER 2018

SPONSOR: GRANTS DANA FUNDAMENTAL PPUKM (FF-2019-300)

RESEARCH TITLE:

DEVELOPMENT AND EFFECTIVENESS OF MYPINKMOM EDUCATIONAL MODULE FOR PREGNANT WOMEN WITH ANAEMIA IN PETALING DISTRICT

RAUDAH BINTI ABD RAHMAN

P92513

RESEARCH PROPOSAL SUBMITTED AS PARTIAL FULFILLMENT FOR DOCTOR OF PUBLIC HEALTH

FACULTY OF MEDICINE

UNIVERSITI KEBANGSAAN MALAYSIA

2018

DECLARATION

I hereby declare that the work in this proposal is my own work except for few quotations which have been acknowledged. There is no conflict of interest in the study team. Investigator who found to have conflict of interest will be excluded in this study.

06 JULY 2018 RAUDAH BINTI ABD RAHMAN

P 92513

TABLE OF CONTENT

|  | | |  | PAGE |
| --- | --- | --- | --- | --- |
| DECLARATION | | |  | i |
| TABLE OF CONTENT | | |  | ii-iv |
| ACKNOWLEDGEMENT | | |  | v |
| CHAPTER 1 | | | INTRODUCTION |  |
| 1.1 | | | Introduction | 1-2 |
| 1.2 | | | Problem Statement | 2-3 |
| 1.3 | | | Research Justification | 3 |
| 1.4 | | | Objectives |  |
|  | | | 1.4.1 General objective | 3 |
|  | | | 1.4.2 Specific objectives | 3-4 |
| 1.5 | | | Research Questions | 4 |
| 1.6 | | | Hypothesis | 4 |
| 1.7 | | | Summary of chapter | 5 |
| CHAPTER 2 | | | LITERATURE REVIEW |  |
| 2.1 | | | Introduction | 6 |
| 2.2 | | | Anaemia in pregnancy | 7 |
| 2.3 | | | Burden of anaemia in pregnancy | 7-8 |
| 2.4 | | | Risk factors of iron deficiency anaemia in pregnancy | 8-10 |
| 2.5 | | | Effects of anaemia in pregnancy | 10-11 |
| 2.6 | | | Intervention studies on anaemia in pregnancy | 11-13 |
| 2.7 | | | Instructional System Design (ISD) Model for educational module development | 13-14 |
| 2.8 | | | Mobile Health (m-health) in educating patients | 14-15 |
| 2.9 | | | Summary of chapter | 15 |
| Figure 1 | Theoretical framework of Health Belief Model | | | 16-17 |
| Figure 2 | Conceptual framework | | | 18-19 |
| CHAPTER 3 | METHODOLOGY | | |  |
| 3.1 | Introduction | | | 20 |
| 3.2 | Part 1: Validation of study instrument | | | 20-21 |
| 3.3 | Part 2: Development of MYPINKMOM educational module using ADDIE model (Table 1) | | | 21-22 |
|  |  | | |  |
| 3.4 | Part 3: Intervention study using the developed educational module | | |  |
|  | 3.4.1 Study design | | | 22 |
|  | 3.4.2 Intervention  Table 2: Outline of MYPINKMOM content | | | 23  23-26 |
|  |  | | |  |
|  | 3.4.3 Study sites | | | 26 |
|  | 3.3.4 Study Duration | | | 26 |
|  | 3.3.5 Target population | | | 26 |
|  | 3.3.6 Study population | | | 27 |
|  | 3.3.7 Sampling frame | | | 27 |
|  | 3.3.8 Inclusion criteria | | | 27 |
|  | 3.3.9 Exclusion criteria | | | 27 |
|  | 3.3.10 Sample size calculation | | | 28-29 |
|  | 3.3.11 Sampling method | | | 29 |
|  | Figure 3: Multistage cluster sampling | | | 29 |
|  | 3.3.12 Study flowchart | | | 30 |
|  | 3.3.13 Study procedure | | | 31 |
|  | 3.3.14 Criteria to suspending or terminating study | | | 32 |
|  | 3.3.15 Criteria for subject withdrawal | | | 32 |
|  | 3.3.16 Procedure of subject withdrawal | | | 32 |
|  | 3.3.17 Study Instrument  3.318 Variables | | | 32 – 35  35 |
|  | 3.4.19 Operational Definition | | | 35-38 |
|  | 3.4.20 Data Analysis | | | 38 |
|  | Table 4: Statistical Analysis | | | 38-40 |
| 3.5 | Ethics Consideration | | | 40-41 |
| CHAPTER 4 | EXPECTED RESULTS | | |  |
| 4.1 | | Introduction | | 42 |
| Table 1 | | Sociodemographic characteristics of respondents | | 43 |
| Table 2 | | Comparison of mean difference of knowledge, attitude, practice score and haemoglobin level of intervention and control group at pre-intervention and post-intervention | | 44 |
| Table 3 | | Comparison of knowledge score on anaemia in pregnancy between intervention and control group | | 45 |
| Table 4 | | Comparison of attitude score on anaemia in pregnancy between intervention and control group | | 46 |
| Table 5 | | Comparison of practice score on anaemia in pregnancy between intervention and control group | | 47 |
| Table 6 | | Comparison of haemoglobin level between intervention and control group | | 48 |
| Table 7 | | Comparison of iron-rich food intake between intervention and control group | | 49 |
| Table 8 | | Comparison of iron-enhancer food intake between intervention and control group | | 50 |
| Table 9 | | Comparison of iron-inhibitor food intake between intervention and control group | | 51 |
| REFERENCES | |  | | 52-55 |
| APPENDIX A | | BUDGET | | 56-57 |
| APPENDIX B | | GANTT CHART | | 58 |
| APPENDIX C | | SOCIODEMOGRAPHIC QUESTIONNAIRES | | 59 |
| APPENDIX D | | FOOD FREQUENCY QUESTIONNAIRES (FFQs) | | 60-64 |
| APPENDIX E | | KNOWLEDGE, ATTITUTE, PRACTICE (KAP) ON IRON DEFICIENCY ANAEMIA IN PREGNANCY QUESTIONNAIRES | | 65-76 |
| APPENDIX F | | INFORMATION TO THE RESPONDENTS | | 77-84 |
| APPENDIX G | | WRITTEN CONSENT FORM | | 85-86 |

ACKNOWLEDGEMENT

Praise to Allah with His permission I am able to accomplish this task in the stipulated time. I would like to express my gratitude to my dedicated supervisors, Assc.Prof. Dr. Idayu Badilla binti Idris and Prof. Dr. Zaleha binti Md Isa for the valuable guidance and support. Special thanks also for Dr Rahana binti Abdul Rahman, an Obstetrics and Gynaecology specialist and lecturer, UKMMC for the expert opinion as well as to all the lecturers in Community Health Department UKMMC. I also want to acknowledge Food Agriculture Organization (FAO) for allowing me to adapt their validated Knowledge, Attitude, Practice (KAP) questionnaire on iron deficiency anaemia and translate it into Malay language. The acknowledgment also goes to Prof. Dr Hamid Jan from Universiti Sains Malaysia for allowing me to adapt the validated Pregnancy Food Frequency Questionnaire (FFQ). Last but not least, special acknowledgement I dedicated to my beloved husband, my family and friends for their encouragement and support.

**CHAPTER 1: INTRODUCTION**

1.1 Introduction

World Health Organization (WHO) has defined anaemia as haemoglobin of less than 13g/dl in men and less than 12g/dl. In pregnant women, haemoglobin less than 11g/dl is considered as anaemia (WHO 2001). It is a major public health problem worldwide especially in developing countries including Malaysia. The most common cause of anaemia in pregnancy is iron deficiency which is defined as anaemia with serum ferritin of less than 15ug/L (ABC of Clinical Haematology 2007). Prevalence of anaemia in pregnancy globally in 2016 was 40.1% (World Bank Data 2016). Even with routine nationwide prophylaxis of haematinics supplement programme in Malaysia, prevalence of anaemia in pregnancy was 33% in urban Selangor ([Soh et al. 2015](#_ENREF_43)), 43.6% in urban Perak ([Mahdy et al. 2017](#_ENREF_26)) and 57.4% in Jerteh, Terengganu ([Nik Rosmawati et al. 2012](#_ENREF_37)).

The association between severe anaemia and maternal death using data from the WHO Multi-countries Survey on maternal health showed that maternal death in women with severe anaemia are two times higher than women without anaemia ([Daru et al. 2018](#_ENREF_10)). Pregnant women with anaemia are associated with high risk of severe bleeding during delivery ([Khaskheli et al. 2016](#_ENREF_23); [Tusimin et al. 2016](#_ENREF_49)), and heart failure ([Khaskheli et al. 2016](#_ENREF_23); [Tangeda et al. 2016](#_ENREF_46)) which can lead to maternal death. Effects to newborn are small for gestational age ([Jusoh et al. 2015](#_ENREF_20); [Tusimin et al. 2016](#_ENREF_49)), low birth weight ([Nair et al. 2016](#_ENREF_35)), prematurity ([Chen et al. 2017](#_ENREF_7); [Jusoh et al. 2015](#_ENREF_20)) and risk of iron deficiency anaemia in newborn which increase perinatal morbidity and mortality ([De Sa et al. 2015](#_ENREF_11); [Mclimore et al. 2013](#_ENREF_27)).

The effectiveness of iron supplementation on pregnant women with iron deficiency anaemia was proven in many intervention studies ([Etheredge et al. 2015](#_ENREF_13); [Hemminki et al. 2016](#_ENREF_17); [Mei et al. 2014](#_ENREF_28); [Mwangi et al. 2015](#_ENREF_34); [Nguyen et al. 2016](#_ENREF_36); [Zhao et al. 2015](#_ENREF_51)). Even so, in reality, the iron supplementation does not seem to prevent maternal anaemia as most of anaemic mothers are found to be non-compliant to the iron tablets ([Gebre et al. 2015](#_ENREF_15); [Sajith et al. 2016](#_ENREF_39); [Titilayo et al. 2016](#_ENREF_48)). Non-compliance is influenced by several factors such as poor knowledge ([Compaore et al. 2014](#_ENREF_9); [Souganidis et al. 2012](#_ENREF_44), ) , forgetfulness and afraid of the side effects of iron tablets ([Arega Sadore et al. 2015](#_ENREF_2); [Gebre et al. 2015](#_ENREF_15); [Titilayo et al. 2016](#_ENREF_48)).

Based on this background, education on anaemia in pregnancy for pregnant women is very important. One of the models frequently used in educational intervention to improve preventive behavior, perception and awareness on disease is Health Belief Model (HBM) ([Hochbaum et al. 1952](#_ENREF_18)). HBM-based education interventions using verbal training were proven to improve knowledge and haemoglobin level of anaemic pregnant women ([Baharzadeh et al. 2017](#_ENREF_3); [Noronha et al. 2013](#_ENREF_38); [Widyawati et al. 2015](#_ENREF_50)).

In view of the growth in smartphone network coverage worldwide, mobile health (m-health) which is delivery of healthcare education or services through mobile telecommunication technologies such as smartphone applications, WhatsApp, or Telegram messaging services are relevant nowadays as according to International Telecommunication Union (ITU). Globally, there are more than 5 billion wireless users and 70% of them are from low to middle income countries (Global Observatory of eHealth, WHO 2011).

This study is aimed to develop MYPINKMOM educational module based on HBM using WhatsApp application as learning tools for anaemic pregnant women. It will be used to evaluate the effect on haemoglobin status, dietary iron intake as well as knowledge, attitude and practice in this group of women. This study will be conducted in urban population in Petaling District, which is the most populated district in Selangor, Malaysia.

1.2 Problem Statement

Despite implementation of nationwide prophylaxis of haematinics supplement programme, the prevalence of anaemia in pregnancy in Malaysia is still high especially among urban population ([Mahdy et al. 2017](#_ENREF_26); [Soh et al. 2015](#_ENREF_43)). Maternal anaemia is caused mainly by low compliance to iron supplementation ([Gebre et al. 2015](#_ENREF_15); [Sajith et al. 2016](#_ENREF_39); [Titilayo et al. 2016](#_ENREF_48)) due to poor knowledge, attitude and practice ([Jose et al. 2016](#_ENREF_19); [Kulkarni 2015](#_ENREF_25)).

1.3 Research Justification

Intervention studies on education intervention using verbal training were proven to improve knowledge and anaemic status of pregnant women with anaemia in many studies ([Baharzadeh et al. 2017](#_ENREF_3); [Noronha et al. 2013](#_ENREF_38); [Widyawati et al. 2015](#_ENREF_50)). However, intervention using mobile health (m-health) based education module for anaemia in pregnancy is still scarce. Therefore, the present study will develop educational module using mobile technology application and evaluate its effectiveness on haemoglobin level as well as knowledge, attitude and practice on iron deficiency anaemia among pregnant women.

1.4 Objectives

1.4.1 General objective:

To develop a new intervention programme using mobile technology namely MYPINKMOM and to evaluate its effectiveness in improving haemoglobin level of pregnant women with anaemia in Petaling district.

1.4.2 Specific objectives:

1. To develop and validate a theory and evidence-based intervention programme using mobile technology to improve haemoglobin level among participants.
2. To develop and validate a questionnaire on knowledge on anaemia in pregnancy
3. To develop and validate a questionnaire on attitude towards adherence to iron supplement
4. To compare sociodemographic characteristics (age, race, education level, and occupation), obstetric profile (gestational week, gravida, and type of iron supplement prescribed), and outcome measurement (haemoglobin level, knowledge on anaemia in pregnancy score, dietary iron intake, dietary iron enhancer (vitamin C) intake, iron inhibitor (tannin, phytate and calcium) intake, and attitude towards adherence to iron supplement (attitude score, subjective norms score, and perceived behaviour control score) between intervention and control group at baseline.
5. To compare mean primary outcome (haemoglobin level) and secondary outcome measurements (knowledge on anaemia in pregnancy score, dietary iron intake, dietary iron enhancer (vitamin C) intake, iron inhibitor (tannin, phytate, and calcium) intake, attitudes towards adherence to iron supplement (attitude score, subjective norms score, and perceived behaviour control score) between baseline and at week 6 (immediately after intervention) within the intervention and the control group.
6. To determine mean difference of primary outcome and secondary outcome measurements between the intervention and the control group at week 6 (immediately after intervention).
7. To determine the changes in primary outcome measurement over time (at baseline, at week 6 (immediately after intervention), and at week 12 (6 weeks after intervention) between the intervention and the control group.

1.5 Research Questions

1. Is the new theory and evidence-based intervention programme using mobile technology to prevent anaemia in pregnancy valid?
2. Is the newly developed Knowledge on Anaemia in Pregnancy Questionnaire valid to measure knowledge on anaemia in pregnancy?
3. Is the newly developed Attitude towards Adherence to Iron Supplement Questionnaire valid to measure attitude towards adherence to iron supplement intake among pregnant women?
4. Does the newly developed MYPINKMOM intervention programme improve haemoglobin level, knowledge on anaemia in pregnancy, dietary iron, iron enhancer and iron inhibitor intake, and attitude towards adherence to iron supplement intake?

1.6 Hypothesis

1. There is no difference in mean sociodemographic characteristics (age, race, education level, occupation), obstetric profile (gestational week, gravida, types of iron supplement prescribed) and outcome measurement (haemoglobin level, knowledge on anaemia in pregnancy score, dietary iron intake, dietary iron-enhancer (vitamin C) intake, iron-inhibitor (tannin, phytate, calcium) intake, attitudes towards adherence to iron supplement (attitude score, subjective norms score, perceived behaviour control score) between intervention and control group at baseline
2. There is a difference in mean haemoglobin level, knowledge on anaemia in pregnancy score, dietary iron intake, dietary iron-enhancer (vitamin C) intake, iron-inhibitor (tannin, phytate, calcium) intake, attitudes towards adherence to iron supplement (attitude score, subjective norms score, perceived behaviour control score) between baseline and at week 6 (immediately after intervention) within the intervention group.
3. There is no difference in mean haemoglobin level, knowledge on anaemia in pregnancy score, dietary iron intake, dietary iron-enhancer (vitamin C) intake, iron-inhibitor (tannin, phytate, calcium) intake, attitudes towards adherence to iron supplement (attitude score, subjective norms score, perceived behaviour control score) between baseline and at week 6 (immediately after intervention) within the control group.
4. There is a difference between haemoglobin level, knowledge on anaemia in pregnancy score, dietary iron intake, dietary iron-enhancer (vitamin C) intake, iron-inhibitor (tannin, phytate, calcium) intake, attitudes towards adherence to iron supplement (attitude score, subjective norms score, perceived behaviour control score) at week 6 (immediately after intervention) between intervention and control group.
5. There is a difference in haemoglobin level over times i.e. at baseline, at week 6 (immediately after intervention) and at week 12 (6 weeks after intervention) between intervention and control group.

1.7 Summary of chapter

Prevalence of anaemia in pregnancy in Malaysia is still high in spite of nationwide prophylaxis of haematinics supplement to pregnant women. Pregnant women with anaemia are associated with poor compliance to haematinics supplement and poor knowledge, attitude and practice on anaemia in pregnancy. Therefore, education intervention is important. The effectiveness of educational intervention using verbal training is proven. However, in view of increasing usage of wireless and smartphone technology globally, online health educational intervention using mobile smartphone technology (mobile health or m-health) is more relevant nowadays, yet it is still scarce. Therefore, this study will develop an educational module using smartphone technology application which is WhatsApp application. Health Belief Model will be used as underpinning theoretical framework in this educational module.

**CHAPTER 2: LITERATURE REVIEW**

2.1 Introduction

The most common cause of anaemia in pregnancy is iron deficiency which is defined as anaemia with serum ferritin of less than 15ug/L (ABC of Clinical Haematology 2007). Despite nationwide haematinics supplement given to pregnant women in Malaysia, the prevalence of anaemia in pregnancy is still high ([Nik Rosmawati et al. 2012](#_ENREF_37); [Soh et al. 2015](#_ENREF_43); Mahdy et al.2017). In view of increased risk of maternal and perinatal morbidity and mortality associated with anaemia in pregnancy, many studies were conducted to determine risk factors for high prevalence of this public health problem ([Al-Farsi et al. 2011](#_ENREF_1); [Jusoh et al. 2015](#_ENREF_20); [Khaskheli et al. 2016](#_ENREF_23); [Tusimin et al. 2016](#_ENREF_49)). Non-compliance to iron tablets and improper dietary intake were found to be associated with iron deficiency anaemia which is the most common anaemia in pregnancy ([Gebre et al. 2015](#_ENREF_15); [Sajith et al. 2016](#_ENREF_39); [Titilayo et al. 2016](#_ENREF_48)). Poor knowledge and awareness on iron deficiency anaemia among pregnant women were the main reasons why they did not comply to iron supplement and did not practice proper dietary intake ([Compaore et al. 2014](#_ENREF_9)). In view of the high prevalence of iron deficiency anaemia in pregnancy, many intervention studies were conducted to counter this problem. In this literature review, the burden, risk factors, effects, intervention studies on iron deficiency anaemia in pregnancy and Instructional System Design (ISD) Model for educational module development will be explored from multiple published and unpublished studies using online database of journals and reports.

2.2 Anaemia in pregnancy

Anaemia in pregnancy occurs when there is reduction in oxygen capacity of the blood due to a decrease in circulating red blood cells or a decrease in the level of haemoglobin to meet physiological demands of pregnant women. Physiological demands vary, depending on age, gender, altitude (stay above sea-level area), smoking status and trimester of pregnancy (WHO 2011). According to the WHO, anaemia among pregnant women is classified into mild, moderate and severe when haemoglobin level is between 10.0 to 10.9 g/dl, 7.0 to 9.9 g/dl and lower than 7.0 g/dl respectively (WHO 2011). The commonest cause of anaemia in pregnancy is iron deficiency which is characterised by serum ferritin level of less than 15ug/L and haemoglobin level less than 11g/dl among pregnant women (ABC of Clinical Haematology 2007).

In iron deficient women, haemoglobin levels can decline dramatically during pregnancy to compensate for the increasing maternal blood volume and the iron demand of the growing fetus. Maternal haemoglobin level usually drops during the first trimester. Further drop in haemoglobin concentration occurs in the second trimester, and usually start to increase slightly in the third trimester. WHO has not recommended on the use of different haemoglobin cut-off points for anaemia by trimester, but it is recognized that the haemoglobin concentrations decreased by approximately 5 g/l in second trimester (World Health Organization 2011).

2.3 Burden of anaemia in pregnancy

Anaemia is a public health problem globally especially in developing countries. For the purpose of public health, the WHO has classified it based on the prevalence of anaemia among pregnant women. There is no significant public health problem if the prevalence of anaemia is 4.9% or less. It becomes mild, moderate and severe if the prevalence of anaemia is between 5.0% to 19.9%, 20.0% to 39.9% and 40.0% or more respectively (World Health Organization 2011).

Prevalence of anaemia in pregnancy globally in 2016 was 40.1% (World Bank Data 2016). A systematic analysis using health and nutrition survey data from WHO’s Vitamin and Nutrition Information System from 107 countries and summary statistics from agencies showed the prevalence of anaemia among pregnant women in year 2011 was 22% in high income countries, 24% in central and eastern Europe, 25% in east and southeast Asia, 31-56% in Africa, and 27-31% in Latin America ([Stevens et al. 2013](#_ENREF_45)).

In developed countries, the prevalence of anaemia in pregnancy mainly iron deficiency anaemia is relatively low. The prevalence is only 4-5% in Denmark ([Milman 2015](#_ENREF_31)) and 2.5% in Portugal ([Gomes Da Costa et al. 2016](#_ENREF_16)). The prevalence is still high in developing countries even with policy of iron supplement programme implementation in most of the countries. The prevalence of iron deficiency anaemia in pregnancy in Northwest Ethiopia, Africa is 16.6% ([Melku et al. 2014](#_ENREF_29)) and 27.5% in China ([Shao et al. 2012](#_ENREF_41)). In Malaysia, the prevalence was 33% in urban Selangor (Soh et al. 2015), 43.6% in urban Perak ([Mahdy et al. 2017](#_ENREF_26)) and 57.4% in Jerteh, Terengganu ([Nik Rosmawati et al. 2012](#_ENREF_37)).

2.4 Risk factors of anaemia in pregnancy

During pregnancy, there is an increase in iron requirement to increase red cell mass, to expand plasma volume and to allow growth of fetal-placenta unit ([Miller 2013](#_ENREF_30)). These physiological changes are exacerbated by low pre-pregnancy iron stores, increased iron demand during pregnancy, and inadequate dietary iron intake. In view of this, pregnant women become the most vulnerable group to develop anaemia especially due to iron deficiency during pregnancy ([Miller 2013](#_ENREF_30)).

Low iron stores in pre-pregnancy is due to monthly menstrual blood loss and low dietary iron intake. The prevalence of female adolescents in Malaysia with anaemia was 32% and out of this, 88% were iron deficiency anaemia ([Foo et al. 2004](#_ENREF_14)). A study done showed mean dietary iron intake among young women of reproductive age in Malaysia was only 10 mg/day which was below the recommended level of 20-29 mg/day by the Ministry of Health ([Foo et al. 2004](#_ENREF_14)). The Malaysian Clinical Practice Guideline, “The Management of Anaemia in Pregnancy and Chronic Kidney Disease” recommends iron supplement of 100 mg elemental ferrous iron per day for prophylaxis of iron deficiency anaemia in pregnant women. When these women become pregnant, iron stores will deplete further as iron demands increase drastically during pregnancy. ([Miller 2013](#_ENREF_30)).

Pregnant women with low socioeconomic status, high parity ([Khaskheli et al. 2016](#_ENREF_23)) and late antenatal booking ([Jusoh et al. 2015](#_ENREF_20)) are at high risk of having iron deficiency anaemia. The prevalence of lower serum ferritin level among pregnant women in Malaysia is higher among Indians and significantly associated with grand multipara, late antenatal booking (in the third trimester) and lower education level (Mahdy et al. 2017).

Studies done in several developing countries revealed pregnant women with anaemia mostly did not comply with iron supplement ([Gebre et al. 2015](#_ENREF_15); [Sajith et al. 2016](#_ENREF_39); [Thirukkanesh &Zahara 2010](#_ENREF_47); [Titilayo et al. 2016](#_ENREF_48)) and did not practice proper dietary intake ([Milman 2015](#_ENREF_31)). In Malaysia, 50.8% of the pregnant women did not comply with iron supplement ([Thirukkanesh&Zahara 2010](#_ENREF_47)). The prevalence of anaemia was higher among these women ([Sajith et al. 2016](#_ENREF_39); [Thirukkanesh&Zahara 2010](#_ENREF_47)). However, there was no significant difference in compliance or adherence rate of iron supplement between urban and rural area ([Gebre et al. 2015](#_ENREF_15); [Thirukkanesh&Zahara 2010](#_ENREF_47)). Younger mother, stays at urban area, higher education level, higher socioeconomic status and attending antenatal clinic as early as in first trimester were the factors associated with increased compliance rate of iron supplement ([Titilayo et al. 2016](#_ENREF_48)).

Low adherence to iron supplement and improper iron dietary intake are mainly due to low knowledge and awareness on iron deficiency anaemia, forgetfulness, afraid of side effects of iron and misperception of fetal macrosomia due to the iron intake ([Arega Sadore et al. 2015](#_ENREF_2); [Gebre et al. 2015](#_ENREF_15)). A qualitative study on knowledge and perception of iron supplementation among pregnant women found that their understanding on anaemia was having “little” blood and only a few felt that they were at high risk of getting anaemia during pregnancy, and giving iron can increased blood volume and strengthened resistance to infection rather than increased red blood cell ([Compaore et al. 2014](#_ENREF_9)). Pregnant women with iron deficiency anaemia was also found to be associated with poor knowledge, attitude and practice on the disease (Jose et al. 2016; Kulkarni 2015). In Indonesia, Souganidis et al. (2012) revealed that maternal knowledge on anaemia was poor but there was no significant association between maternal knowledge of anaemia and maternal level of haemoglobin ([Souganidis et al. 2012](#_ENREF_44)).

2.5 Effects of anaemia in pregnancy

Pregnant women with anaemia commonly presented with symptoms like reduced effort tolerance and cognitive functions which impaired their quality of life. Anaemia in pregnancy especially in the third trimester will cause increased risk of complications of severe bleeding at delivery. Antepartum and postpartum bleeding which are strongly associated with iron deficiency anaemia ([Khaskheli et al. 2016](#_ENREF_23); [Tusimin et al. 2016](#_ENREF_49)) is a major cause of maternal death worldwide (WHO, Maternal Mortality 2016). In a study done in a university hospital in India, 305 pregnant women had iron deficiency anaemia and they were associated with antepartum haemorrhage (16.06%), renal failure (15.73%), disseminated intravascular coagulation (17.1%) and maternal death (5.2%) ([Khaskheli et al. 2016](#_ENREF_23)). Another study also demonstrated a significant association between the severity of anaemia among pregnant women with post-partum haemorrhage (p=0.012) ([Tusimin et al. 2016](#_ENREF_49)).

The association between severe anaemia and maternal death using data from the WHO Multi-country Survey on maternal and newborn health from Latin America, Africa, the Western Pacific, eastern Mediterranean, and southeast Asia showed the maternal death in pregnant women with severe anaemia is 2 times higher than those without severe anaemia ([Daru et al. 2018](#_ENREF_10)). A review of observational studies showed with each 10 g/L increase in maternal haemoglobin, maternal mortality rate was reduced by 29% (OR 0·71 [95% CI 0·60–0·85]) ([Black et al. 2013](#_ENREF_4)).

Maternal serum ferritin level was found to be significantly and positively correlated with newborn serum ferritin level (r=0.389, p=0.01) ([De Sa et al. 2015](#_ENREF_11)). Another study in China also showed significant but weak positive correlation between maternal serum ferritin and newborn cord blood ferritin level (r=0.07, p<0.0001) ([Shao et al. 2012](#_ENREF_41)). This indicates that newborn iron status is dependent on their mother’s iron status during pregnancy.

Iron is important for brain development of fetus and newborn. Therefore, iron deficiency anaemia impaired fetal brain development and psychomotor development of the newborn. A 2-year prospective study which compared mental development index at 12, 18 and 24 months old babies to mothers with anaemia in pregnancy and normal haemoglobin during pregnancy showed the developmental index was significantly lower among babies of anaemic mothers compared to non-anaemic mothers ([Chang et al. 2013](#_ENREF_6)).

Newborn to mothers with anaemia were found to have high risk of small for gestational, low birth weight and prematurity ([Jusoh et al. 2015](#_ENREF_20)) which is associated with increased perinatal mortality. There was significant association between severity of anaemia among pregnant women and small for gestational age (p=0.004) ([Tusimin et al. 2016](#_ENREF_49)). While in another study found that there was a positive correlation between maternal haemoglobin level and low birth weight among newborn (r=0.26, p=0.04) ([Samimi et al. 2012](#_ENREF_40)).

However, maternal haemoglobin and ferritin level were not significantly correlated with anthropometric parameters of the newborn (weight, height and head circumference) ([De Sa et al. 2015](#_ENREF_11); [Samimi et al. 2012](#_ENREF_40)). Yet, poor maternal iron status can lead to higher infant fibroblast growth factor-23 (FGF23) and phosphate level in infants which cause bone mineralisation disorders such as hereditary hypophosphatemic rickets ([Braithwaite et al. 2016](#_ENREF_5)).

2.6 Intervention studies on anaemia in pregnancy

In view of its negative effects to mothers and newborn which increase maternal morbidity and mortality, many intervention studies were carried out to solve this public health problem. Intervention studies on anaemia in pregnancy especially due to iron deficiency are mostly conducted as clinical trials in community.

A clinical trial was done in Mozambique which evaluated the maternal and child effects between two groups i.e routine prophylactic iron supplement group and those who were anaemic and treated. The results showed both groups had low prevalence of preterm delivery, low birth weight, perinatal deaths and women’s death during pregnancy or postpartum ([Hemminki et al. 2016](#_ENREF_17)). A double-blinded randomised controlled trial (RCT) by Zhao et al (2014) on 1632 non-anaemic pregnant women, which evaluated anaemic status in intervention group who received iron and folate supplement, and in control group who received only folate supplement showed significant increase in haemoglobin level in intervention group. Similar outcome also seen in other double-blinded RCT. Etheredge et al (2015) compared iron versus placebo and found significant difference of mean haemoglobin between intervention and control group. Mwangi et al (2015) who compared zinc and iron versus placebo showed increment in haemoglobin level among patients receiving zinc. Mei et al (2014) and Nguyen et al (2016) who compared folate only versus folate and iron versus folate, iron and micronutrient showed increment of haemoglobin in both groups who receiving folate and iron, and folate, iron and micronutrient. All of these intervention studies showed iron supplementation was associated with improvement in anaemic status ([Etheredge et al. 2015](#_ENREF_13); [Mei et al. 2014](#_ENREF_28); [Mwangi et al. 2015](#_ENREF_34); [Nguyen et al. 2016](#_ENREF_36); [Zhao et al. 2015](#_ENREF_51)).

Besides clinical trials, education intervention study was also conducted. For example, a new educational model was introduced in Indonesia in managing iron deficiency anaemia in pregnancy. This study found that there was increment of haemoglobin level among study subjects in intervention group who received the educational intervention as compared to control group ([Widyawati et al. 2015](#_ENREF_50)). Another intervention study in India which empowered pregnant women to take appropriate action to overcome iron deficiency anaemia based on the education intervention given on iron deficiency anaemia, dietary modification and proper iron supplement intake resulted in significant improvement in maternal haemoglobin level in intervention group when compared to control group ([Noronha et al. 2013](#_ENREF_38)). A randomised controlled trial study was performed on 80 pregnant women in Iran in which the patients in experimental group who received two educational sessions showed increase in knowledge and awareness score on iron deficiency anaemia ([Baharzadeh et al. 2017](#_ENREF_3)). These educational intervention studies used Health Belief Model (HBM) as theoretical framework for their educational module which is the most frequently used theory in health education in preventing disease. HBM was first established in 1950s to investigate why tuberculosis screening programme by US Public Health Service failed that time ([Hochbaum et al. 1952](#_ENREF_18)). Later, it was extended by Leventhal, Rosenstock and others to explain influence of individual perceptions towards disease and how these perceptions influence behaviour that affect compliance or adherence to treatment.

In Health Belief Model, the likelihood of individual to change behaviour in preventing disease is mainly influenced by their decision in considering the costs and benefits of action based on their perception on the disease. The likelihood of action or behavioural change is influenced by perceived susceptibility, severity, benefits to action, barriers to action and a stimulus or cue to action such as change in health, doctor’s advice, family or friends’ death that trigger action for behavioural change to adhere to treatment (Hochbaum et al.1952).

2.7 Instructional System Design (ISD) Model for educational module development

Instructional Systems Design model (ISD) is frequently used for development of educational module. The origin of Instructional System Design (ISD) Model is obscure, but the underlying concepts of ISD can be traced to the model developed for the United States armed forces in the mid-1970s. The Centre for Educational Technology at Florida State University worked with the U.S. Army to develop a model, which evolved into the Interservice Procedures for Instructional Systems Development (IPISD), intended for the Army, Navy, Air Force, and Marine Corps. Branson provides a graphic overview of the IPISD, which shows five top-level headings: analyse, design, develop, implement, and control (ADDIC) ([Molenda 2003](#_ENREF_32)).

ISD is a systematic procedure that includes steps of analysing, designing, developing, implementing and evaluating instruction to improve the quality and effectiveness of learning. The model is goal-directed whereby it guides the preparation of instruction to achieve objective. Each step in this model is interdependence and emphasizes the congruency among objectives, instruction and evaluation. Instructional theories also play an important role in the design of instructional materials. Theories such as behaviourism, constructivism, social learning and cognitivism help shape and define the outcome of instructional materials ([Kruse 2002](#_ENREF_24)).

The most frequently used ISD by instructional designers and training developers is the ADDIE model. The model includes five phases (Analysis, Design, Development, Implementation, and Evaluation) which represent a dynamic, flexible guideline for building effective training and educational materials. In the ADDIE model, each step has an outcome that feeds into the subsequent step ([Kruse 2002](#_ENREF_24); [Molenda 2003](#_ENREF_32)).

Other ISD models include the Dick & Carey model ([Dick 1996](#_ENREF_12)) and Morrison-Ross-Kemp model ([Kemp et al. 2007](#_ENREF_21); [Morrison et al. 2010](#_ENREF_33)). In Dick & Carey model, four core concepts are emphasized, which are interculturalism, interrelatedness, inquiry and pedagogical leadership ([Dick 1996](#_ENREF_12)). In Morrison-Ross-Kemp model, nine elements are emphasised; instructional problems, learner characteristics, task analysis, instructional objectives, content sequencing, instructional strategies, designing the message, instructional delivery and evaluation instrument ([Morrison et al. 2010](#_ENREF_33)).

2.8 Mobile Health (m-health) in educating patients

In view of continued growth of smartphone network coverage worldwide, mobile health (m-health) which is defined as delivery of healthcare education or services through application of mobile telecommunication technologies, has potential for healthcare delivery services transformation including health promotion (WHO 2011). Example of delivery of m-health are Smartphone applications, WhatsApp, or Telegram messaging services which are relevant nowadays as according to International Telecommunication Union (ITU). Globally there are more than 5 billion wireless subscribers and 70% are from low to middle income countries (WHO 2011).

The emerging of m-health concept represents the evolution of electronic health (e-health) systems with the use of the internet for healthcare as well as mobile applications with internet connectivity (WHO 2011). The study and development of m-health services and applications have been a focus of the researchers nowadays. There are a number of intervention studies using m-health for education of patients, such as development of educational smartphone application for coronary artery disease (CAD) patients in South Korea ([Cho et al. 2014](#_ENREF_8)), and Health Belief Model based educational module on awareness on cervical cancer screening using telegram instant messaging services in Iran ([Khademolhosseini et al. 2017](#_ENREF_22)). Besides, m-health is also essential for monitoring, prevention of disease and assists in diagnosis in more advanced settings ([Silva et al. 2015](#_ENREF_42)). Besides, m-health services are even becoming popular in developing countries where healthcare facilities are frequently remote and inaccessible ([Silva et al. 2015](#_ENREF_42)). However, there is no study on m-health based educational module for anaemia in pregnancy published.

2.9 Summary of chapter

Anaemia in pregnancy is a public health problem especially in developing countries as it is associated with high maternal and perinatal mortality and morbidity. Most of anaemia in pregnancy is due to iron deficiency. Pregnant women with untreated anaemia are at high risk of severe bleeding during delivery and heart failure due to anaemia that can eventually cause maternal death. Effect to newborn are low birth weight, prematurity, impaired brain development and risk of anaemia especially due to iron deficiency. Pregnant women with low socio-economic status, high parity, late antenatal booking and poor birth spacing are at high risk of getting anaemia. Poor knowledge, attitude and practice among pregnant women with anaemia cause low compliance to iron supplementation and improper iron dietary intake which are the two reasons why prevalence of iron deficiency anaemia in pregnancy is still high. Therefore, it is important to explore knowledge, attitude and practice on anaemia as well as education intervention to solve this public health problem. The effectiveness of educational based intervention on anaemia in pregnancy using Health Belief Model are proven. However, most of the educational intervention were using traditional way of health education which were talk, booklets and posters. The emerging of mobile health (m-health) is relevant for educating patients nowadays. For effective and quality educational module development, Instructional System Design Model such as ADDIE model is frequently used.

Figure 1: Theoretical framework of Health Belief Model

Health Belief Model (Figure 1) which will be used as theoretical framework for educational module in this study is the most frequently used theory in health education in preventing disease. It was first established in 1950s to investigate why tuberculosis screening programme by US Public Health Service failed that time ([Hochbaum et al. 1952](#_ENREF_18)). Later, it was extended by Leventhal, Rosenstock and others to explain influence of individual perceptions towards disease and how these perceptions influence behaviour that affect compliance or adherence to treatment.

In Health Belief Model, the likelihood of individual to take action and change behaviour to prevent the disease is influenced by their decision in balancing the costs and benefits of action taken based on their perception on the disease. The likelihood of action like compliance with treatment is influenced by perceived susceptibility (perception of the chances of getting the disease), perceived severity (perception of the severity or seriousness of the disease to the person when he or she get the disease), perceived benefits of an action (perception that the action that will be taken is effective in reducing the health effect), perceived barriers to action (perception of what difficulties if proposed action taken) and a stimulus or cue to action such as change in health, doctor’s advice, family or friends’ death can trigger action to change behaviour to adhere with treatment.

Perceived susceptibility and perceived severity of the disease which are called perceived threat is also modified by factors like sociodemographic (age, ethnicity, gender, socioeconomic status), personality and knowledge level of the disease.

Figure 2: Conceptual framework

The conceptual framework is shown in Figure 2. From literature review, anaemia in pregnancy can cause increase risk of maternal mortality and morbidity as well as perinatal mortality and morbidity. Factors that influencing anaemia in pregnancy can be divided into distal factors and proximal factors. Distal factor is defined as risk factor that represents an underlying vulnerability for a particular condition or event. Distal factors that influencing anaemia in pregnancy are socioeconomic status, demographic (age, race), poor obstetric history (late antenatal booking, multiparity and poor spacing) and poor pre-pregnancy iron status. Proximal factor is defined as factors in the immediate situation that influence thoughts, feelings, or behaviour of individuals. In this study, the proximal factors for anaemia in pregnancy are dietary habit, compliance to treatment, knowledge, attitude and practice on anaemia in pregnancy. Public health and educational intervention conducted usually focusing on these proximal factors.

CHAPTER 3: METHODOLY

- - 1. Introduction

This chapter elaborates on how the study will be conducted. This study is divided into three parts. Part 1 consists of the development and validation of the newly developed intervention programme, namely the MYPINKMOM intervention programme. This intervention programme is developed using the ADDIE Model. Part 2 comprises of the development and validation of two study instruments, that are Knowledge on Anaemia in Pregnancy Questionnaire and Attitude towards Adherence to Iron Supplement Questionnaire, which are based on TPB. These questionnaires are developed to measure the effectiveness of the MYPINKMOM intervention programme.

Finally, Part 3 includes a quasi-experimental study design measuring the effectiveness of this intervention programme. In this final part of the study, primary outcome measurement is haemoglobin level. Secondary outcome measurements are knowledge score on anaemia in pregnancy, dietary iron, dietary iron enhancer, and iron inhibitor intake, as well as attitude towards adherence to iron supplement based on TPB construct. For this quasi-experimental study, the study design, study location, study duration, eligibility criteria, sample size calculation, sampling method, blinding, data collection, data analysis plan, and ethics consideration are elaborated as well. Primary outcome (haemoglobin level) is measured at baseline and at week 6 (immediately after intervention). Secondary outcomes (knowledge level on anaemia in pregnancy, dietary iron, vitamin C, tannin, phytate and calcium intake, attitude score, subjective norms score, and perceived behaviour score on adherence to iron supplement intake are measured at baseline and at week 6 (immediately after intervention).

- - 1. Part 1: Development and validation of the MYPINKMOM intervention programme

The MYPINKMOM intervention programme is conducted and validated using the ADDIE (Analysis, Design, Development, Implementation, and Evaluation) model, which is a systematic structural design model, commonly used for the development of educational programme or intervention (Kruse 2002). This intervention programme is developed in five phases that are analysis, design, development, implementation, and evaluation phases, according to this model.

- - - 1. Analysis phase

During the analysis phase, literature reviews, clinical guidelines, and existing educational pamphlets aimed at giving information to pregnant women with anaemia are analysed and reviewed. Subsequently it will be used as evidence-based material to be included in the development of the MYPINKMOM intervention programme. The content of the MYPINKMOM intervention programme is formulated and discussed among three expert panels which consist of a Public Health Physician, an Obstetrician & Gynaecologist Specialist, and a Nutritionist who were selected based on their expertise in prevention and health promotion programme for anaemia, clinical management for anaemia in pregnancy, and nutritional management for anaemia in pregnancy, respectively.

- - - 1. Design phase

Consequently, in the design phase, the learning objectives of MYPINKMOM intervention programme is outlined, the content was defined, and the specific intervention in each part, and the method of dissemination of the information is established. Furthermore, in this phase, the evaluation methods for the validation and the actual study for the intervention programme are also designed.

Based on the findings from the analysis phase and HBM construct, the learning objectives for this mobile technology-based intervention programme are outlined which are as follows: 1) To deliver basic knowledge on anaemia in pregnancy, and causes and complications of anaemia in pregnancy among pregnant women, 2) To demonstrate the correct way of iron supplement and dietary intakes to improve anaemic status among pregnant women, 3) To demonstrate the correct way to overcome possible side effects of iron intake that might inhibit compliance to iron supplement among pregnant women.

The MYPINKMOM intervention programme is divided into six parts or components that are presented via six video clips and disseminated through WhatsApp application to pregnant women. The outline of this intervention programme content for each part is designed based on the learning objectives. For each part in MYPINKMOM intervention programme, the outlines of the content are based on HBM construct and learning objectives as drawn as in Table 3.1.

| **MYPINKMOM intervention programme** | **Health Belief Model construct** | **Objective** | **Outline of MYPINKMOM intervention programme content** |
| --- | --- | --- | --- |
| Part 1 (Video 1) | Perceived susceptibility | To educate participants on general information about anaemia in pregnancy. | Explanation about haemoglobin and its function.  Definition of anaemia  The common causes of anaemia in pregnancy. |
| Part 2 (Video 2) | Perceived severity | Complications of untreated anaemia in pregnancy | Explanation on the effects of untreated anaemia in pregnancy to mothers and newborn. |
| Part 3 (Video 3) | Perceived susceptibility | To explain about the pathophysiology of anaemia in pregnancy and method of anaemia detection. | Explanation on the pathophysiology that render pregnant women to be high risk for anaemia.  Explanation on the signs and symptoms of anaemia. |
| Part 4 (Video 4) | Perceived benefit | To educate participants on the nutrition required for pregnant women with anaemia | Information regarding sources of iron in food.  Information regarding food that enhances and prevent iron absorption in the body. |
| Part 5 (Video 5) | Perceived benefit | To educate participants on iron supplement therapy and correct method of iron supplement intake | Information regarding the different types of iron supplement available in the market.  Information regarding the correct method of iron tablet intake. |
| Part 6 (Video 6) | Perceived barrier | To educate participants on possible side effects of oral iron tablets and methods to overcome it. | Explanation about the possible side effects of iron tablet.  Explanation about the steps to overcome the side effects. |

During this phase, we will also design the evaluation methods to determine the validation of the MYPINKMOM intervention programme and to measure the effectiveness of this intervention programme in the subsequent actual intervention study.

**Development phase**

As mentioned above, based on the findings from the design phase, six parts or components of the mobile technology-based intervention, namely MYPINKMOM intervention programme is initially developed in the form of PowerPoint slide presentations. The PowerPoint slides are converted into video clips with interesting infographic, animation and audio explanation designed by a graphic computer designer.

**Implementation phase**

In the implementation phase or pilot study of the MYPINKMOM intervention programme, all six videos are shown to five experts consisting of two Public Health Physicians, two Nutritionists, and an Obstetrics & Gynaecology Specialist and to five pregnant women attending a government antenatal clinic in Petaling Jaya.

**Evaluation phase**

The aim of the evaluation phase is to validate the newly developed MYPINKMOM intervention programme. In this evaluation phase, all six video clips are shown to five experts for assessment of the content validity. In this study, Content Validity Index (CVI) is used to determine content validity of videos in MYPINKMOM intervention programme. CVI is being widely used for the assessment of content validity of instrument that is conducted by a panel of experts (Polit et al. 2007; Polit et al. 2006). All experts and pregnant women who are involved in the implementation phase also give comments on the presentation of the video clips.

**Part 2: Development and validation of study instruments**

**Adaptation and Validation of the** **Questionnaire on Knowledge regarding Anemia in Pregnancy**

The items of Questionnaire on Knowledge regarding Anaemia in Pregnancy are adapted from the knowledge section of Nutrition-Related KAP questionnaire model produced by Food and Agriculture Organization (FAO) of United Nation (Marías & Glasauer 2014). Permission is obtained from the author. The items are modified based on MYPINKMOM intervention programme content.

A total of 30 items related to knowledge on anaemia in pregnancy is adapted and modified. The responses for each item were “Yes”, “No”, and “Not Sure”. One point is given to correct answer and zero point is given to incorrect and “Not Sure” answer. The items are all in Malay and English language.

Subsequently, content validity and face validity of the Questionnaire on Knowledge regarding Anaemia in Pregnancy is performed. Pilot testing is performed for evaluation of internal consistency of the questionnaire.

**Content validity**

Content validity is the assessment of the validity of the content of each item to identify whether the sample of items represents domains of the scale in the instrument (Polit et al. 2006). A panel of experts who are well-versed on the job or field of interest in the test or questionnaire will judge the existence of “overlap between the test and the job performance domain” (Lawshe 1975). In this study, three experts who consist of a Public Health Physician, an Obstetrics & Gynaecologist Specialist, and a Nutritionist determined the content validity of each item. Each item will be rated by each expert as “essential”, “useful but not essential”, or “not necessary” for appropriateness, accuracy, and ambiguity of each item. Content validity of each item is determined by calculating Content Validity Ratio (CVR) using Lawshe’s formula (Lawshe 1975). According to Lawshe (1975), if the number of experts is five and less, only item with CVR of at least 0.99 is retained. In other words, only items that received “essential” rate from all experts are retained if the number of experts involved is five or less. The CVR formula is as follows:

| $\mathrm{CVR} = n - (N/2) / (N/2)$ |  |
| --- | --- |

Where,

CVR = content validity ratio for each item

n = number of expert rate particular item as “essential”

N = total number of experts

**Face validity**

After performing content validity, back-to-back translation that is translation from English to Malay and subsequently to English again of the retained items was performed. This is essential, as the questionnaire was initially prepared in English. It was performed by two independent individuals who are healthcare providers and are able to speak and write well in English and Malay. Then, face validity was carried out by eight participants consisted of doctors to assess comprehensibility of the retained items in the questionnaire. Face validity is a subjective evaluation of items in study instrument or questionnaire, which is usually conducted by a group of individuals that have knowledge on the matter (Setia 2017). Corrections are done based on the comments given.

**Reliability**

Pilot testing will be done on 150 pregnant women with anaemia attending one government antenatal clinic in Petaling Jaya, Malaysia. Item-to-subject ratio (five items: one subject) is used to determine the minimum sample size required for the validation of this questionnaire (Hair, Black, et al. 1998). Internal consistency is evaluated by determining Cronbach alpha value for all items in the questionnaire. The acceptable Cronbach alpha is at least 0.70 (Hair, Black, et al. 1998).

**Development and** **validation of the Questionnaire on Attitudes towards Adherence to Iron Supplement**

**Development of the questionnaire**

Unlike the previous questionnaire that is adapted from an existing questionnaire, this questionnaire is developed based on TPB. The purpose of this questionnaire was to assess participants’ attitude towards adherence to iron supplement based on TPB construct, which is able to predict participants’ intention and likelihood to adhere or comply to iron supplement throughout their pregnancy.

The development of this questionnaire is carried out using a guideline in constructing TPB-based questionnaire (Ajzen 2002, 2006). According to this guideline, the first step is to determine the action or behaviour of interest by defining the elements of Target, Action, Context, and Time (TACT) (Ajzen 2002). In this study, the behaviour of interest is “adherence to daily iron supplement intake in pregnancy”. In this case, “iron supplement intake” is the Context, “adherence” is the Action, “daily” is the Target, while “in pregnancy” is the Time.

The intention to adhere to iron supplement every day throughout pregnancy is determined by behavioural belief, normative belief, and control belief towards adherence to iron supplement (Ajzen & Processes 1991). Behavioural belief will usually determine attitude, which refers to favourable or unfavourable belief towards adherence to iron supplement. Meanwhile, normative belief regulates subjective norm which refers to social pressure, expectation, and motivation by other people, which influence pregnant women to comply to iron supplement. Meanwhile, control belief determined perceived behaviour control that is defined as self-efficacy or ability to adhere to daily iron supplement (Ajzen 1985; Ajzen 2002; Ajzen & Processes 1991).

Before formulating items for each construct in this questionnaire, a survey using open-ended questionnaire is conducted on 15 pregnant women from one antenatal clinic in Petaling Jaya to elicit behaviour belief, normative belief and control belief towards adherence to daily iron supplement (Ajzen 2002, 2006). The questions to elicit participants’ behaviour belief are on advantages and disadvantages of adherence to iron supplement. Meanwhile, the questions to elicit participants’ normative belief are on whether people around the pregnant women motivate or demotivate them to take daily iron tablet, while the questions for control belief are on the factors that they think can enable or stop them to adhere to iron tablet intake every day. The list of open-ended questions in the questionnaire is shown in Table 3.2.

Table 3.2 Content of open-ended questions to elicit commonly held beliefs on compliance to iron supplement

| Objective | Questions |
| --- | --- |
| To elicit behavioural belief towards adherence to daily iron supplement throughout pregnancy | What do you believe in terms of the advantages of adherence to daily iron supplement intake during pregnancy?  What do you believe in terms of the disadvantages of adherence to daily iron supplement intake during pregnancy? |
| To elicit normative belief towards adherence to daily iron supplement throughout pregnancy | What do people surrounding you do that motivate you to adhere to daily iron supplement?  What do people surrounding you do that demotivate you to adhere to daily iron supplement? |
| To elicit control belief towards adherence to daily iron supplement throughout pregnancy | What factors that increase your ability to adhere to iron tablet intake every day?  What factors that impede your ability to adhere to iron tablet intake every day? |

The findings will be elaborated in Chapter IV (Results). Based on these findings, literature reviews along with discussions with the expert panels, 18 items are developed. Responses for each item are interval scale ranging from 1 to 7, in which “1” reflects extremely disagree and “7” reflects extremely agree (Ajzen 2006). The items are developed in Malay and English languages.

**Pretesting**

Pretesting of the newly developed questionnaire consists of content validity and face validity.

**Content validity**

Three appointed experts, determined the content validity of each item. Each item will be rated by each expert as “essential”, “useful but not essential”, or “not necessary” for appropriateness, accuracy, and ambiguity of each item. Similar to the previous validation for Questionnaire on Knowledge regarding Anaemia in Pregnancy, content validity of each item is determined by calculating CVR using Lawshe’s formula (Lawshe 1975) as follows:

| $\mathrm{CVR} =\frac{n - \left( \frac{N}{2} \right)}{\left( \frac{N}{2} \right)}$ |  |
| --- | --- |

Where,

CVR = content validity ratio

n = number of experts involved

N = number of experts who rated particular item as “essential”

Since the number of experts involved are five or less, only items with CVR of at least 0.99 or items received “essential” rating from all experts are retained (Lawshe 1975).

Face validity

The content validity will be followed by face validity that is performed by eight participants who consisted of healthcare staff who can speak and write well in both English and Malay, for comprehensibility of the questionnaire (Miller & Lovler 2018). Corrections such as replacement of jargon and ambiguous words are done based on the comments from the participants.

- - - - 1. Pilot test

As this questionnaire is newly developed, a pilot test will be conducted on 100 pregnant women from an antenatal clinic in Petaling Jaya who are on iron supplement and self-reported that they are compliant to the iron supplement. This pilot test is performed to evaluate the psychometric properties, which is Exploratory Factor Analysis (EFA) and internal consistency (Cronbach alpha value) of the items in the questionnaire. Confirmatory Factor Analysis (CFA) for validation of the items in the questionnaire is also subsequently performed on another 100 pregnant women from an antenatal clinic in Petaling Jaya. Item-to-subject ratio of five to one is used to determine the minimum sample size required for the validation of this questionnaire (Hair, Anderson, et al. 1998).

- - 1. Part 3: Intervention study using MYPINKMOM intervention programme
       1. Study location

The study is conducted in Petaling district, in Selangor, Malaysia. Petaling is the most highly populated district in Selangor. According to the latest official Population Census conducted by the Department of Statistics Malaysia in 2010, the population in Petaling district (excluding foreigners) is 1.661 million people recorded, where 51.2% are males and 48.8% are females (Population Census of Malaysia 2010). As for ethnicity, 52.6% are Malays, 34.9% are Chinese, 11.5% are Indians, and 1% are others (Population Census of Malaysia 2010). Majority (73.9%) are at reproductive age between 15 - 64 years old, while 22.5% are aged 65 years old and more, and 3.6% are below 14 years old (Population Census of Malaysia 2010).

Petaling is divided into three cities based on the local government jurisdictions that are Petaling Jaya, Subang Jaya, and Shah Alam. Petaling is also divided into 5 *mukim,* which are Bandar Petaling Jaya, Bukit Raja, Damansara, Petaling, and Sungai Buloh.

**Study design**

A two-arm cluster-assignment, single-blinded, randomized controlled trial (RCT) with a 1:1 allocation ratio will be conducted in health clinics and groups with participants (pregnant women with anemia) in the Petaling district, which is one of the most populated districts in Selangor, Malaysia. In this study, two government antenatal clinics in Petaling district will be randomly selected as study sites. Each antenatal clinic is located in Petaling Jaya and in Subang Jaya respectively, and they are 20 km apart. The antenatal clinic in Petaling Jaya will be randomly assigned as intervention group while the other clinic in Subang Jaya will be assigned as the control group. Pregnant women from the government antenatal clinic in Petaling Jaya will be recruited as intervention group, while pregnant women from the government antenatal clinic in Subang Jaya will be recruited as control group. This is performed to avoid possible contamination of information between respondents from the same clinic.

**Study duration**

The study will be held for 2 years 6 months, from September 2017 until mid-March 2020. This duration includes preparation of proposal, ethical approval, and validation of study instruments, and development, implementation, and evaluation of MYPINKMOM intervention programme.

The preparation of proposal and ethical approval will take place from September 2017 until January 2019. Subsequently, the development of MYPINKMOM intervention programme and the validation of both study instruments will be performed from February 2019 until October 2019. Finally, the intervention programme will be conducted from November 2019 until mid-March 2020.

**Target population**

The study population is pregnant women with anaemia who attended government antenatal clinics in Petaling district.

**Study sample**

Study sample are pregnant women who attended the two government antenatal clinics, diagnosed with anaemia (haemoglobin level of less than 11 g/dl), and fulfilled the inclusion criteria.

**Inclusion criteria**

The inclusion criteria are pregnant women at 13 - 24 gestational week, aged 20 - 40 years old, diagnosed with anaemia with haemoglobin level of 7.0 - 10.9 g/dl, able to read and write in Malay and/or English, as well as owning a smartphone with WhatsApp application installed.

**Exclusion criteria**

The exclusion criteria are pregnant women who are diagnosed with anaemia due to thalassemia, diagnosed with other haematological diseases, renal failure or cancer, and/or have history of symptomatic anaemia (breathlessness, chest pain, syncope, or signs of heart failure) in this current pregnancy and/or planned for termination of pregnancy and/or received parenteral iron and/or blood transfusion throughout intervention period.

**Matching**

The participants from intervention and control groups are matched for age (20 - 40 years old), gestational week (13 - 24 weeks of gestation) and haemoglobin level (7.0 - 10.9 g/dl) at recruitment.

**Sampling frame**

Sampling frame is the list of pregnant women who attended the selected government antenatal clinics in Petaling district and who are diagnosed with anaemia with haemoglobin level of 7.0 - 10.9 g/dl.

**Sampling unit**

Primary sampling unit is Petaling district. Secondary sampling unit is cities in Petaling district, namely Petaling Jaya and Subang Jaya. Tertiary sampling unit is government antenatal clinics in each selected city. Final sampling unit is pregnant women who attended the selected government antenatal clinic.

**Sample size calculation**

The sample size is calculated using the following formula for clinical trial comparing mean of two groups (Sakpal 2010).

Formula of calculating sample size is:

| $n = \frac{[{(Z_{\alpha/2}+Z_{\beta})}^{2} \times{(ó1 + ó2)}^{2}]}{{(\mu1 - \mu2)}^{2}}$ |  |
| --- | --- |

Where,

n = sample size per arm

μ1 = mean haemoglobin of intervention group after intervention in previous study i.e. 11.97g/dL (Senanayake et al. 2010).

μ2 = mean haemoglobin of control group after intervention in previous study i.e. 11.1g/dL (Senanayake et al. 2010).

μ1-μ2 = difference of mean haemoglobin for intervention and control group after intervention i.e. 11.97-11.1= 0.87 (Senanayake et al. 2010).

ó1 = standard deviation for intervention group i.e. 0.9 (Senanayake et al. 2010)

ó2 = standard deviation for control group i.e. 1.3 (Senanayake et al. 2010).

Z_α/2_ =1.96 with significance level of 5%

Z_β_ = 0.84 with power of 80%

For Z_α/2_, Z is a constant value according to the accepted α error and whether it is a one-sided or two-sided effect as shown below:

Table 3.3 Constant value according to α error

| **α error** | **5%** | **1%** | **0.1%** |
| --- | --- | --- | --- |
| 2-sided | 1.96 | 2.5758 | 3.2905 |
| 1-sided | 1.65 | 2.33 |  |

Source: Sakpal 2010

For Z1-β, Z is a constant value according to the power of the study as shown below:

| Power | 80% | 85% | 90% | 95% |
| --- | --- | --- | --- | --- |
| Value | 0.8416 | 1.0364 | 1.2816 | 1.6449 |

Source: Sakpal 2010

Therefore, the sample size for this study when calculated is:

n=50 per arm. Total sample size was 120 including 20% drop out.

Sampling method

The sampling method that is selected in this study is multistage random sampling. Petaling district is initially selected using purposive sampling. Out of the three cities in Petaling district, as mentioned above, Petaling Jaya and Subang Jaya are randomly selected using computer generated random numbers.

Next, one government antenatal clinic from each selected city will be randomly selected using computer generated random numbers. One of the selected clinics will be randomly assigned as the intervention site. Meanwhile, another clinic will be assigned as the control site. A total of 60 pregnant women that are diagnosed with anaemia and fulfilled the eligibility criteria from the clinic that is randomly selected as intervention site will be randomly selected to be the intervention group. Meanwhile, 60 pregnant women with anaemia and fulfilled the eligibility criteria from the clinic that is randomly selected as control site will be randomly selected to be the control group.

Allocation

Pregnant women who attended both selected antenatal clinics will be screened for inclusion and exclusion criteria. Participants who fulfilled the eligibility criteria will be consented and baseline assessment will be done on the same day. Subsequently, 60 participants who fulfilled the inclusion criteria from each selected antenatal clinic will be randomly selected. The selected participants from the clinic that is randomly assigned as intervention site will be allocated as the intervention. Meanwhile, those from the clinic that is assigned as control site will be allocated as the control group.

Blinding

No blinding in this study. The participants and investigators are aware of the intervention received by the participants.

Study variables

- - - - 1. Dependent variables

Dependent variables are:

1. Haemoglobin level at week 6 (immediately post intervention) and at week 12 (6 weeks post intervention)
2. Knowledge scores on anaemia in pregnancy at week 6 (immediately post intervention)
3. Attitude towards adherence to iron supplement using TPB construct (attitude score, subjective norms score, perceived behaviour control score) at week 6 (immediately post intervention)
4. Dietary iron, iron enhancer (vitamin C) and iron inhibitor (tannin, phytate, calcium) intake at week 6 (immediately post intervention)
   - - - 1. Independent variables

Independent variables are:

1. Types of intervention received (intervention or control)
2. Sociodemographic characteristics (age, race, education level, occupation)
3. Obstetric history (gestational week, gravida, types of iron supplement received)
4. Outcome measurements (haemoglobin level, knowledge scores on anaemia in pregnancy, attitude towards adherence to iron supplement using TPB construct, and dietary iron, vitamin C, tannin, phytate and calcium intake) at baseline

3.4.16 Operational definition of variables

Operational definition for each dependent and independent variable are as follows:

a. Dependent variables:

i. Haemoglobin level post intervention

Haemoglobin level that is obtained from Full Blood Count (FBC) during antenatal check-up during antenatal check-up at week 6 (immediately post intervention) and at week 12 (6 weeks post intervention)

ii. Knowledge score on anaemia in pregnancy post intervention

Total score of knowledge on anaemia in pregnancy that is obtained by the participants after answering Questionnaire on Knowledge regarding Anaemia in Pregnancy at week 6 (immediately post intervention).

iii. Attitude score towards adherence to iron supplement post intervention

Attitude towards adherence to iron supplement is determined by TPB construct namely attitude, subjective norms and perceived behaviour control (Ajzen 1985). It reflects the intention or commitment to adhere to daily iron supplement throughout pregnancy (Ajzen 2002). In this study, attitudes towards adherence to iron supplement refers to mean attitude score, mean subjective norms score and mean perceived behaviour control score that are obtained by the participants after answering Questionnaire on Attitude towards Adherence to Iron Supplement for adherence to iron supplement at week 6 (immediately post intervention).

iv. Dietary iron intake post intervention

Dietary iron intake refers to the amount of dietary iron-rich food, iron-enhancer and iron-inhibitor intake in mg/day which is calculated from the Food Frequency Questionnaires for Pregnant Women that will be answered by the participants at week 6 (immediately post intervention).

Sources of dietary iron-rich food are haem-iron such as meat, poultry, fish, seafood, and non-haem iron such as dark green vegetables, legumes, eggs. Iron-enhancer are ascorbic acid or vitamin C rich fruits and certain vegetables such as oranges, limes, guava, and potatoes. Iron-inhibitors are food and beverages that contain phytate (cereal breakfast, oat, rice), phenol (coffee, tea, cocoa), tannin (tea, coffee) and calcium (milk, soy, cheese) (Recommended Nutrient Intakes for Malaysia, 2017).

b. Independent variables:

i. Age

Refers to the age of participants at baseline assessment.

ii. Race

Refers to the ethnicity of participants.

iii. Education level

Refers to the highest education level that participants have.

iv. Occupation

Refers to the participants’ job during study period.

v. Gravida

The number of pregnancies the woman had including the current pregnancy.

Parity

The number of children that has been delivered regardless alive or not.

Gestational week

The gestational week at baseline assessment.

Types of iron supplement

The type of iron supplement that is prescribed to participants by doctor in charge during current pregnancy. The examples are Zincofer, Maltofer, Ferrous, Iberet, or Obimin.

Group of participants

Refers to group of participants either intervention or control group, according to types of intervention they received during the study period.

Haemoglobin level at baseline

Haemoglobin level that is obtained from Full Blood Count (FBC) during antenatal check-up during antenatal check-up before intervention.

Knowledge score on anaemia in pregnancy at baseline

Total score of knowledge on anaemia in pregnancy that is obtained by the participants after answering Questionnaire on Knowledge regarding Anaemia in Pregnancy before intervention.

Attitude score towards adherence to iron supplement post intervention

Attitude towards adherence to iron supplement refers to mean attitude score, mean subjective norms score, and mean perceived behaviour control score of TPB construct that are obtained by the participants after answering Questionnaire on Attitudes towards Adherence to Iron Supplement before intervention.

Dietary iron intake post intervention

Dietary iron intake refers to the amount of dietary iron-rich food, iron enhancer and iron inhibitor intake in mg/day calculated from the FFQs for Pregnant Women that is answered by the participants before intervention.

Study intervention

Participants in the intervention group received MYPINKMOM intervention programme which is disseminated through WhatsApp application. Meanwhile, participants from the control group received information on anaemia that is available in their antenatal book. The description of MYPINKMOM intervention for intervention group and the intervention for control group is shown in Table 3.5.

Table 3.5 Comparison of intervention methods between intervention and control group

|  | **Intervention group** | **Control group** |
| --- | --- | --- |
| Intervention | MYPINKMOM intervention programme consists of 6 video clips with info-graphic and audio explanation, followed by weekly reminder on proper dietary and iron supplement intake | Explanation on anaemia in pregnancy by researcher using information available in participants’ antenatal book |
| Method of dissemination of information | Through WhatsApp application | Researcher gave explanation on anaemia in pregnancy in front of the participant |
| Duration /timeline | One video clip delivered daily for 6 days in Week 1, followed by weekly reminder at Week 2-5 | The information delivered once during baseline assessment |
| Theory-based | Using Health Belief Model as foundation in the development of MYPINKMOM intervention programme | Not known |
| Weekly reminder | Given.  The weekly reminder given will be given as follow:  “Salam Puan. How are you? Thank you for watching all the 6 videos. You are encouraged to watch these videos frequently and practice the tips on proper dietary and iron supplement intake as in the videos. You may ask me for any inquiry if any”.  “*Salam Puan, Apa khabar? Terima kasih kerana telah menonton keenam-enam video tersebut. Puan adalah digalakkan untuk menonton video-video tersebut berulang-ulang kali dan, mengamalkan tips pemakanan dan pengambilan ubat tambah darah yang betul seperti di dalam video tersebut. Jika ada sebarang kemusykilan, Puan boleh ajukan kepada saya*” | Not given |
| Iron supplement | Participants are on iron supplement as prescribed by the doctor in charge during their routine antenatal visits. | Participants are on iron supplement as prescribed by the doctor in charge during their routine antenatal visits. |

MYPINKMOM intervention program

MYPINKMOM intervention program is a newly developed and validated intervention programme on anaemia in pregnancy. It consists of 6 video clips (Part 1-6) with the duration of 3 - 5 minutes each. The videos will be delivered to participants of the intervention group through the WhatsApp application daily at week 1. The information on anaemia in pregnancy will be delivered in the form of interesting infographics and clear audio explanations. The videos will be presented in the Malay language. The content for each video is shown in Appendix A - F.

From week 2 to week 5, participants in intervention group will receive weekly reminder through the WhatsApp application to repeatedly go through the videos and, apply proper dietary and iron supplement intake as shown in the videos. This will be an interactive session as participants are also encouraged to ask questions on anaemia in pregnancy.

- - - 1. Participant’s timeline

Eligibility screening is carried out before the enrolment and allocation of participants. The total duration of participation for each respondent in this study was 12 weeks. Pre-test or baseline assessment is held at the beginning of week 1 before the intervention commenced and after consent from participants will be obtained. The intervention is carried out at week 1 till week 5. Post-intervention test is subsequently carried out at week 6 and at week 12. Participants’ timeline is shown in Table 3.6.

Table 3.6 Participant’s timeline for the intervention group

|  | Study period | | | | | | | | | | | | | | | | | | | |  |
| --- | --- | --- | --- | --- | --- | --- | --- | --- | --- | --- | --- | --- | --- | --- | --- | --- | --- | --- | --- | --- | --- |
|  | Enrolment | | Post enrolment | | | | | | | | | | | | | | | | | |  |
| Time point (week) | 0 | | 1 | | 2 | | 3 | | 4 | | | 5 | | | 6 | | | 12 | | |  |
|  | I | C | I | C | I | C | I | C | | I | C | | I | C | | I | C | | I | C | |
| Eligibility screen | **√** | √ |  |  |  |  |  |  | |  |  | |  |  | |  |  | |  |  | |
| Informed consent | √ | √ |  |  |  |  |  |  | |  |  | |  |  | |  |  | |  |  | |
| **Pre-test** | | | | | | | | | | | | | | | | | | | | |  |
| Haemoglobin level |  |  | √ | √ |  |  |  |  | |  |  | |  |  | |  |  | |  |  | |
| Knowledge score on Anaemia in Pregnancy |  |  | √ | √ |  |  |  |  | |  |  | |  |  | |  |  | |  |  | |
| Attitudes on Adherence to Iron Supplement |  |  | √ | √ |  |  |  |  | |  |  | |  |  | |  |  | |  |  | |
| Dietary iron, vitamin C and caffeine intake |  |  | √ | √ |  |  |  |  | |  |  | |  |  | |  |  | |  |  | |
| **Intervention** | | | | | | | | | | | | | | | | | | | | |  |
| Explanation using information from antenatal book |  |  | - | √ |  |  |  |  | |  |  | |  |  | |  |  | |  |  | |
| Video part 1 |  |  | √ | - |  |  |  |  | |  |  | |  |  | |  |  | |  |  | |
| Video part 2 |  |  | √ | - |  |  |  |  | |  |  | |  |  | |  |  | |  |  | |
| Video part 3 |  |  | √ | - |  |  |  |  | |  |  | |  |  | |  |  | |  |  | |
| Video part 4 |  |  | √ | - |  |  |  |  | |  |  | |  |  | |  |  | |  |  | |
| Video part 5 |  |  | √ | - |  |  |  |  | |  |  | |  |  | |  |  | |  |  | |
| Video part 6 |  |  | √ | - |  |  |  |  | |  |  | |  |  | |  |  | |  |  | |
| Weekly reminder |  |  |  |  | √ | - | √ | - | | √ | - | | √ | - | |  |  | |  |  | |
| **Post-test** |  |  |  |  |  |  |  |  | |  |  | |  |  | | √ | √ | | √ | √ | |
| Haemoglobin level |  |  |  |  |  |  |  |  | |  |  | |  |  | |  |  | |  |  | |
| Knowledge score |  |  |  |  |  |  |  |  | |  |  | |  |  | | √ | √ | |  |  | |
| Attitudes on Adherence to Iron Supplement |  |  |  |  |  |  |  |  | |  |  | |  |  | | √ | √ | |  |  | |
| Dietary iron, vitamin C and caffeine intake |  |  |  |  |  |  |  |  | |  |  | |  |  | | √ | √ | |  |  | |

“I” indicates intervention group, “C” indicates control group, “√” indicates done,” - “indicates not done

- - - 1. Data collection methods

Participants who agreed to join the intervention study give their informed consent in an isolated room in the clinic. Data collection will be performed during their antenatal checkup visit. Baseline assessments are conducted before carrying out the intervention. Meanwhile, post-intervention assessments are conducted at week 6 and at week 12 (Table 3.7).

Table 3.7 Data collection method

| **Data collection** | **Time** | **Outcome measurements** |
| --- | --- | --- |
| Baseline assessment | Week 1  (before intervention) | - Sociodemographic characteristics (age, race, educational level, occupation) and Obstetric history (gravida, parity, gestational week, booking hemoglobin, types of iron supplement prescribed) using Sociodemographic and Obstetric Profile Questionnaire - Hemoglobin level (Full Blood Count) traced from antenatal book - Dietary iron intake (iron-rich food, iron-enhancer, iron-inhibitor) that is calculated from the amount of food intake for the past month using Food Frequency Questionnaire for pregnant women - Knowledge score on anaemia in pregnancy using Questionnaire on Knowledge regarding Anemia in Pregnancy - Attitudes score towards adherence to iron supplement intake using TPB construct (attitudes, subjective norms, perceived behaviour control score) using Questionnaire on Attitudes towards Adherence to Iron Supplement |
| Post-intervention assessment | Week 6 (immediately after intervention) | - Hemoglobin level (Full Blood Count) traced from antenatal book - Dietary iron intake (iron-rich food, iron-enhancer, iron-inhibitor) that is calculated from the amount of food intake for the past month using Food Frequency Questionnaire for pregnant women - Knowledge scores on anemia in pregnancy using Questionnaire on Knowledge regarding Anemia in Pregnancy - Attitudes score towards adherence to iron supplement intake using TPB construct (attitudes, subjective norms, perceived behavior control score) using Questionnaire on Attitudes towards Adherence to Iron Supplement |
| Post-intervention assessment | Week 12  (6 weeks after intervention) | - Hemoglobin level (Full Blood Count) traced from antenatal book |

- - - 1. Consort diagram

Consort participants diagram of the study flow is illustrated as in Figure 3.3, below.

Enrollment

Assessed for eligibility

Excluded

Analysed (n=)
 Excluded from analysis (n=)

Analysed (n=)
 Excluded from analysis (n=)

Analysis

Loss to follow up / Discontinued intervention

Follow up at week 12

Loss to follow up/ Discontinued intervention

Loss to follow up/ Discontinued intervention

Follow up at week 6

Randomisation

Loss to follow up/ Discontinued intervention

Allocated as control group (n=60)

• Received allocated intervention (n=60)

Allocated as intervention group (n=60)

• Received allocated intervention (n=60)

Allocation

**Data Analysis**

The data will be initially analysed descriptively, and normality of continuous data is determined. The sociodemographic characteristics (age, race, educational level, occupation), obstetric history (gravida, parity, gestational week, booking haemoglobin, types of iron supplement prescribed) and haemoglobin level, dietary iron intake, knowledge score on anaemia in pregnancy and attitude score towards adherence to iron supplement intake at baseline are compared between intervention and control group using Student t-test and Chi-squared test.

The mean difference of haemoglobin level, knowledge score on anaemia in pregnancy, attitude score towards adherence to iron supplement intake, dietary iron-rich food, iron-enhancer and iron-inhibitor intake between baseline and at week 6 (immediately post intervention) within both intervention and control group are subsequently compared using Paired t-test.

The mean difference of haemoglobin level, knowledge score on anaemia in pregnancy, attitude score towards adherence to iron supplement intake, dietary iron-rich food, iron-enhancer and iron-inhibitor intake at week 6 (immediately post intervention) between intervention and control group, as well as the effect size of the intervention for each dependent variable (haemoglobin level, knowledge score on anaemia in pregnancy, attitude score towards adherence to iron supplement intake, dietary iron-rich food, iron-enhancer and iron-inhibitor intake) will be measured using Multivariate Analysis of Variance (MANOVA).

Subsequently the changes of haemoglobin level over time i.e. at baseline, at week 6 (immediately post intervention) and at week 12 (6 weeks post intervention) between intervention and control group will be determined using Repeated Measures ANOVA. Level of significance was predetermined at p-value<0.05. In this study, only participants who completed the study at week 6 and at week 12 will be included for the analysis. This gives an estimate of true efficacy of the intervention i.e. the effect of the intervention among the participants who completed the intervention as planned (Ranganathan et al. 2016).

ETHICS CONSIDERATION

The study will be ethically conducted according to the Declaration of Helsinki. Participation in this study is voluntary. The participants will be given adequate time to read the Patient Information Sheet and Written Informed Consent (Appendix N). All patients are briefly explained about the study by the researcher and they are allowed to ask any inquiry. Patients who agreed to participate in the study signed the consent form in an isolated room in the clinic.

Participants’ confidentiality will be always preserved throughout the study and during presentation and publication of this study. No identification data will be revealed in the questionnaire, during presentation, and publication later in future. The participants shall not be given access to their personal information and study data. However, the participants can be informed about this research findings if they wish to know. Data will be kept for five years before it will be destroyed.

REFERENCES

ABC of Clinical Haematology 2007

American Society of Haematology

Al-Farsi, Y. M., Brooks, D. R., Werler, M. M., Cabral, H. J., Al-Shafei, M. A. & Wallenburg, H. C. 2011. Effect of High Parity on Occurrence of Anemia in Pregnancy: A Cohort Study. *BMC Pregnancy Childbirth* 11(7.

Arega Sadore, A., Abebe Gebretsadik, L. & Aman Hussen, M. 2015. Compliance with Iron-Folate Supplement and Associated Factors among Antenatal Care Attendant Mothers in Misha District, South Ethiopia: Community Based Cross-Sectional Study. *J Environ Public Health* 2015(781973.

Baharzadeh, K., Marashi, T., Saki, A., Zare Javid, A. & Araban, M. 2017. Using of Health Belief Model to Promote Preventive Behaviors against Iron Deficiency Anemia among Pregnant Women. *Journal of Research and Health* 7(2): 754-762.

Black, R. E., Victora, C. G., Walker, S. P., Bhutta, Z. A., Christian, P., De Onis, M., Ezzati, M., Grantham-Mcgregor, S., Katz, J. & Martorell, R. 2013. Maternal and Child Undernutrition and Overweight in Low-Income and Middle-Income Countries. *The lancet* 382(9890): 427-451.

Braithwaite, V., Prentice, A., Darboe, M., Prentice, A. & Moore, S. 2016. The Effects of Maternal Iron Deficiency on Infant Fibroblast Growth Factor-23 and Mineral Metabolism. *Bone* 83(1-8.

Chang, S., Zeng, L., Brouwer, I. D., Kok, F. J. & Yan, H. 2013. Effect of Iron Deficiency Anemia in Pregnancy on Child Mental Development in Rural China. *Pediatrics* 131(3): e755-e763.

Chen, K.-J., Chang, Y.-L., Chang, H., Su, S.-Y., Peng, H.-H., Chang, S.-D. & Chao, A.-S. 2017. Long-Term Outcome of Pregnancy Complicating with Severe Aplastic Anemia under Supportive Care. *Taiwanese Journal of Obstetrics and Gynecology* 56(5): 632-635.

Cho, M. J., Sim, J. L. & Hwang, S. Y. 2014. Development of Smartphone Educational Application for Patients with Coronary Artery Disease. *Healthcare informatics research* 20(2): 117-124.

Compaore, A., Gies, S., Brabin, B., Tinto, H. & Brabin, L. 2014. "There Is Iron and Iron..." Burkinabe Women's Perceptions of Iron Supplementation: A Qualitative Study. *Matern Child Health J* 18(8): 1976-1984.

Daru, J., Zamora, J., Fernández-Félix, B. M., Vogel, J., Oladapo, O. T., Morisaki, N., Tunçalp, Ö., Torloni, M. R., Mittal, S. & Jayaratne, K. 2018. Risk of Maternal Mortality in Women with Severe Anaemia During Pregnancy and Post Partum: A Multilevel Analysis. *The Lancet Global Health*

De Sa, S. A., Willner, E., Duraes Pereira, T. A., De Souza, V. R., Teles Boaventura, G. & Blondet De Azeredo, V. 2015. Anemia in Pregnancy: Impact on Weight and in the Development of Anemia in Newborn. *Nutr Hosp* 32(5): 2071-2079.

Dick, W. 1996. The Dick and Carey Model: Will It Survive the Decade? *Educational Technology Research and Development* 44(3): 55-63.

Etheredge, A. J., Premji, Z., Gunaratna, N. S., Abioye, A. I., Aboud, S., Duggan, C., Mongi, R., Meloney, L., Spiegelman, D. & Roberts, D. 2015. Iron Supplementation in Iron-Replete and Nonanemic Pregnant Women in Tanzania: A Randomized Clinical Trial. *JAMA pediatrics* 169(10): 947-955.

Foo, L. H., Khor, G. L., Tee, E. S. & Prabakaran, D. 2004. Iron Status and Dietary Iron Intake of Adolescents from a Rural Community in Sabah, Malaysia. *Asia Pac J Clin Nutr* 13(1): 48-55.

Gebre, A., Mulugeta, A. & Etana, B. 2015. Assessment of Factors Associated with Adherence to Iron-Folic Acid Supplementation among Urban and Rural Pregnant Women in North Western Zone of Tigray, Ethiopia: Comparative Study. *International Journal of Nutrition and Food Sciences* 4(2): 161-168.

Gomes Da Costa, A., Vargas, S., Clode, N. & L, M. G. 2016. Prevalence and Risk Factors for Iron Deficiency Anemia and Iron Depletion During Pregnancy: A Prospective Study. *Acta Med Port* 29(9): 514-518.

Hemminki, E., Nwaru, B. I., Salomé, G., Parkkali, S., Abacassamo, F., Augusto, O., Cliff, J., Regushevskaya, E., Dgedge, M., Sousa, C. & Chilundo, B. 2016. Is Selective Prenatal Iron Prophylaxis Better Than Routine Prophylaxis: Final Results of a Trial (Profeg) in Maputo, Mozambique. *BMJ Open* 6(6):

Hochbaum, G., Rosenstock, I. & Kegels, S. 1952. Health Belief Model. *United States Public Health Service*

Jose, S., Antony, S. C. & Issac, B. 2016. Impact of Knowledge, Attitude and Practice on Anemia Status among Women in Coastal Kochi, Kerala. *Int. J. of Multidisciplinary and Current Research* 4(

Jusoh, N., Ismail, T. a. T. & Daud, A. 2015. Anemia among Teenage Pregnancy in Northwestern Malaysia: What Are the Factors? *INTERNATIONAL JOURNAL OF COLLABORATIVE RESEARCH ON INTERNAL MEDICINE & PUBLIC HEALTH* 7(12): 196-205.

Kadam, P., & Bhalerao, S. (2010). Sample size calculation. International journal of Ayurveda research, 1(1), 55-7.

Kemp, R., Loorbach, D. & Rotmans, J. 2007. Transition Management as a Model for Managing Processes of Co-Evolution Towards Sustainable Development. *The International Journal of Sustainable Development & World Ecology* 14(1): 78-91.

Khademolhosseini, F., Noroozi, A. & Tahmasebi, R. 2017. The Effect of Health Belief Model-Based Education through Telegram Instant Messaging Services on Pap Smear Performance. *Asian Pacific journal of cancer prevention: APJCP* 18(8): 2221.

Khaskheli, M.-N., Baloch, S., Sheeba, A., Baloch, S. & Khaskheli, F. K. 2016. Iron Deficiency Anaemia Is Still a Major Killer of Pregnant Women. *Pakistan Journal of Medical Sciences* 32(3): 630-634.

Kruse, K. 2002. Introduction to Instructional Design and the Addie Model. *Retrieved January* 26(2005.

Kulkarni, K. K. 2015. Kap Studies among Indian Antenatal Women: Can We Reduce the Incidence of Anemia? *The Journal of Obstetrics and Gynecology of India* 65(5): 320-322.

Mahdy, Z., Jumaida, A., Za'im, M. S., Rahana, A., Mukudan, K. & Zaleha, M. 2017. Antenatal Iron Deficiency in an Urban Malaysian Population. *MEDICINE AND HEALTH-KUALA LUMPUR* 12(1): 27-33.

Mclimore, H. M., Phillips, A. K., Blohowiak, S., Pham, D. Q.-D., Coe, C. L., Fischer, B. A. & Kling, P. J. 2013. Impact of Multiple Prenatal Risk Factors on Newborn Iron Status at Delivery. *Journal of pediatric hematology/oncology* 35(6): 473.

Mei, Z., Serdula, M. K., Liu, J.-M., Flores-Ayala, R. C., Wang, L., Ye, R. & Grummer-Strawn, L. M. 2014. Iron-Containing Micronutrient Supplementation of Chinese Women with No or Mild Anemia During Pregnancy Improved Iron Status but Did Not Affect Perinatal Anemia. *The Journal of nutrition* 144(6): 943-948.

Melku, M., Addis, Z., Alem, M. & Enawgaw, B. 2014. Prevalence and Predictors of Maternal Anemia During Pregnancy in Gondar, Northwest Ethiopia: An Institutional Based Cross-Sectional Study. *Anemia* 2014(

Miller, J. L. 2013. Iron Deficiency Anemia: A Common and Curable Disease. *Cold Spring Harbor perspectives in medicine* 3(7): a011866.

Milman, N. 2015. Iron Deficiency and Anaemia in Pregnant Women in Malaysia–Still a Significant and Challenging Health Problem. *J Preg Child Health* 2(168): 2.

Molenda, M. 2003. In Search of the Elusive Addie Model. *Performance improvement* 42(5): 34-37.

Morrison, G. R., Ross, S. M., Kemp, J. E. & Kalman, H. 2010. *Designing Effective Instruction*. John Wiley & Sons.

Mwangi, M. N., Roth, J. M., Smit, M. R., Trijsburg, L., Mwangi, A. M., Demir, A. Y., Wielders, J. P., Mens, P. F., Verweij, J. J. & Cox, S. E. 2015. Effect of Daily Antenatal Iron Supplementation on Plasmodium Infection in Kenyan Women: A Randomized Clinical Trial. *Jama* 314(10): 1009-1020.

Nair, M., Choudhury, M. K., Choudhury, S. S., Kakoty, S. D., Sarma, U. C., Webster, P. & Knight, M. 2016. Association between Maternal Anaemia and Pregnancy Outcomes: A Cohort Study in Assam, India. *BMJ global health* 1(1): e000026.

Nguyen, P. H., Young, M., Gonzalez-Casanova, I., Pham, H. Q., Nguyen, H., Truong, T. V., Nguyen, S. V., Harding, K. B., Reinhart, G. A. & Martorell, R. 2016. Impact of Preconception Micronutrient Supplementation on Anemia and Iron Status During Pregnancy and Postpartum: A Randomized Controlled Trial in Rural Vietnam. *PloS one* 11(12): e0167416.

Nik Rosmawati, N., Mohd Nazri, S. & Mohd Ismail, I. 2012. The Rate and Risk Factors for Anemia among Pregnant Mothers in Jerteh Terengganu, Malaysia. *J Community Med Health Educ* 2(150): 2161-0711.1000150.

Noronha, J. A., Bhaduri, A., Bhat, H. V. & Kamath, A. 2013. Interventional Study to Strengthen the Health Promoting Behaviours of Pregnant Women to Prevent Anaemia in Southern India. *Midwifery* 29(7): e35-41.

Sajith, M., Nimbargi, V., Shah, S., Tekawade, S., Agiwale, J. & Pawar, A. 2016. Correlations of Adherence to Iron Supplements and Prevalence of Anemia in Antenatal Women. *International Journal of Reproduction, Contraception, Obstetrics and Gynecology* 5(10): 3448-3452.

Samimi, M., Asemi, Z., Taghizadeh, M., Azarbad, Z., Rahimi-Foroushani, A. & Sarahroodi, S. 2012. Concentrations of Serum Zinc, Hemoglobin and Ferritin among Pregnant Women and Their Effects on Birth Outcomes in Kashan, Iran. *Oman Medical Journal* 27(1): 40-45.

Shao, J., Lou, J., Rao, R., Georgieff, M. K., Kaciroti, N., Felt, B. T., Zhao, Z.-Y. & Lozoff, B. 2012. Maternal Serum Ferritin Concentration Is Positively Associated with Newborn Iron Stores in Women with Low Ferritin Status in Late Pregnancy. *The Journal of nutrition* 142(11): 2004-2009.

Silva, B. M., Rodrigues, J. J., De La Torre Díez, I., López-Coronado, M. & Saleem, K. 2015. Mobile-Health: A Review of Current State in 2015. *Journal of biomedical informatics* 56(265-272.

Soh, K. L., Tohit, E. R. M., Japar, S., Geok, S. K., Ab Rahman, N. B. & Raman, R. A. 2015. Anemia among Antenatal Mother in Urban Malaysia. *Journal of Biosciences and Medicines* 3(03): 6.

Souganidis, E. S., Sun, K., De Pee, S., Kraemer, K., Rah, J.-H., Moench-Pfanner, R., Sari, M., Bloem, M. W. & Semba, R. D. 2012. Relationship of Maternal Knowledge of Anemia with Maternal and Child Anemia and Health-Related Behaviors Targeted at Anemia among Families in Indonesia. *Maternal and child health journal* 16(9): 1913-1925.

Stevens, G. A., Finucane, M. M., De-Regil, L. M., Paciorek, C. J., Flaxman, S. R., Branca, F., Peña-Rosas, J. P., Bhutta, Z. A., Ezzati, M. & Group, N. I. M. S. 2013. Global, Regional, and National Trends in Haemoglobin Concentration and Prevalence of Total and Severe Anaemia in Children and Pregnant and Non-Pregnant Women for 1995–2011: A Systematic Analysis of Population-Representative Data. *The Lancet Global Health* 1(1): e16-e25.

Tangeda, P. R., Patil, S., Shastri, N. & Noorali, S. N. 2016. Maternal Myocardial Performance in Second Trimester of Pregnancy with Iron Deficiency Anaemia. *Journal of Clinical and Diagnostic Research : JCDR* 10(3): CC16-CC18.

Thirukkanesh, S. & Zahara, A. 2010. Compliance to Vitamin and Mineral Supplementation among Pregnant Women in Urban and Rural Areas in Malaysia. *Pakistan Journal of Nutrition* 9(8): 744-750.

Titilayo, A., Palamuleni, M. E. & Omisakin, O. 2016. Sociodemographic Factors Influencing Adherence to Antenatal Iron Supplementation Recommendations among Pregnant Women in Malawi: Analysis of Data from the 2010 Malawi Demographic and Health Survey. *Malawi Medical Journal* 28(1): 1-5.

Tusimin, M., Yazit, A., Zainulddin, N. & Vaiappuri Vsskn, M. N. S. 2016. The Impact of Severity of Antenatal Anaemia on Maternal and Perinatal Outcome in Hospital Serdang, Central Malaysia. *J Preg Child Health* 3(291): 2.

Widyawati, W., Jans, S., Bor, H. H., Van Dillen, J. & Lagro-Janssen, A. L. 2015. The Effectiveness of a New Model in Managing Pregnant Women with Iron Deficiency Anemia in Indonesia: A Nonrandomized Controlled Intervention Study. *Birth* 42(4): 337-345.

World Health Organisation 2011; Global Prevalence of Anemia in Pregnancy

Zhao, G., Xu, G., Zhou, M., Jiang, Y., Richards, B., Clark, K. M., Kaciroti, N., Georgieff, M. K., Zhang, Z. & Tardif, T. 2015. Prenatal Iron Supplementation Reduces Maternal Anemia, Iron Deficiency, and Iron Deficiency Anemia in a Randomized Clinical Trial in Rural China, but Iron Deficiency Remains Widespread in Mothers and Neonates. *The Journal of nutrition* 145(8): 1916-1923.

APPENDIX A: BUDGET

| Subjects | Number of items | Price per unit | Total (RM) |
| --- | --- | --- | --- |
| Development and validation of MYPINKMOM educational module   1. Computer Programmer/Designer fee 2. Handphone 3. Tape recorder 4. Printing of “Knowledge, Attitude and Practice” for validation | 1  1  1  50 respondents x 4 pages | RM 3000  RM 2000  RM 500  200 x RM 0.20 | RM 3000  RM 2000  RM 500  RM 40 |
| Intervention study:   1. Stationary (pen) to be used by the respondents in answering questionnaires during baseline test, 1 week and 4 weeks after intervention 2. Printing for the Information for respondent sheets 3. Printing for the Written Consent 4. Printing for the Sociodemographic, KAP and FFQs questionnaires for at baseline test 5. Printing for the KAP for post intervention test (1 week after intervention) 6. Printing for the KAP and FFQs questionnaires for post intervention test (4 weeks after intervention) 7. Diary | 360 respondents x 3 tests  360 respondents x 7 pages  360 respondents x 2 pages  360 respondents x 18 pages  360 respondents x 13 pages  360 respondents x 17 pages  360 x 1 unit | 360 x 3 x  RM 1  360 x 7 x  RM 0.20  360 x 2 x RM0.20  360 x 18 x RM 0.20  360 x 13 x RM0.20  360 x 17 x RM0.20  360 x RM1.00 | RM 1080  RM 504  RM 144  RM 1296  RM 936  RM 1224  RM360 |
| TOTAL | RM 11,084.00 | | |

APPENDIX B: GANTT CHART

| No | Activity/Item | 2017 2018 2019 2020 | | | | | | | | |
| --- | --- | --- | --- | --- | --- | --- | --- | --- | --- | --- |
|  |  | Sep | Oct | Nov | Dec | Jan  -June | July  -  Dec | Jan -Jun | July  - Dec | Jan |
| Phase 1: Preparation | |  | | |  | | | | | |
| 1 | Proposal Preparation | X | X |  |  |  |  |  |  |  |
| 2 | Proposal submission |  | X |  |  |  |  |  |  |  |
| 3 | Proposal presentation to department and correction |  | X | X | X | X |  |  |  |  |
| 4 | Proposal presentation to UKM Ethics Committee |  |  |  |  | X | X |  |  |  |
| 5 | Approval from Medical Research Ethics Committee via NMRR |  |  |  |  |  | X |  |  |  |
| 6 | Development and validation of study instruments |  |  |  |  |  | X |  |  |  |
| Phase 2: Plan Execution | |  | | |  | | | | | |
| 7 | Educational module development |  |  |  |  |  | X |  |  |  |
| 8 | Intervention study |  |  |  |  |  |  | X |  |  |
| Phase 3: Data Analysis | |  | | |  | | | | | |
| 9 | Data Entry and Analysis |  |  |  |  |  |  | X |  |  |
| 10 | Thesis writing |  |  |  |  |  |  | X | X |  |
| 11 | Thesis submission |  |  |  |  |  |  |  |  | X |
| 12 | Final report submission |  |  |  |  |  |  |  |  | X |

APPENDIX C: HELAIAN MAKLUMAT PESAKIT/ *PATIENT INFORMATION SHEET*

**Tajuk Penyelidikan:**

Pembinaan dan Keberkesanan Modul Pendidikan MYPINKMOM untuk Wanita Hamil dengan Anemia di daerah Petaling

***Research Title:***

*Development and Effectiveness of MYPINKMOM Educational Module for Pregnant Women with Anaemia in Petaling district*

**Latar Belakang Penyelidikan dan Objektif**

Anemia semasa hamil boleh memberi kesan buruk kepada ibu dan bayi. Kajian ini bertujuan untuk mengkaji faktor-faktor anemia semasa kehamilan dan untuk mendidik wanita hamil untuk mencegah dan mengatasi anaemia semasa hamil. Seramai 360 wanita mengandung daripada 6 klinik kesihatan di daerah Petaling akan menyertai kajian ini. Dalam kajian ini, seramai 180 wanita mengandung akan diberikan maklumat tentang anaemia semasa kehamilan melalui WhatsApp menggunakan modul MYPINKMOM selama 3 minggu yang merupakan eksperimen dalam kajian ini. Manakala seramai 180 orang wanita hamil akan diberikan pamphlet tentang anemia semasa kehamilan. Anda akan telibat dalam kajian ini selama 8 minggu sahaja. Bagi yang mendapat maklumat melalui WhatsApp, anda akan mendapat maklumat tentang anemia semasa kehamilan dalam 2 sesi setiap minggu selama 3 minggu dan anda dikehendaki menulis waktu anda telah selesai membaca maklumat tersebut. Bagi yang mendapat pamphlet tentang anemia semasa kehamilan darpda staf klinik, anda dikehendaki membacanya di rumah.

Pada minggu pertama kajian, borang soal selidik akan diberikan semasa hari pemeriksaan antenatal untuk anda jawab. Pada minggu kedua, ketiga dan keempat kajian, maklumat tentang anemia semasa kehamilan akan diberikan melalui WhatsApp atau pamphlet. Pada minggu kelima kajian, anda akan diberikan borang soal selidik pada hari pemeriksaan anatenatal untuk anda jawab. Pada minggu keenam dan ketujuh kajian, tiada maklumat tentang anemia semasa kehamilan akan diberikan. Pada minggu kelapan kajian, anda akan diberikan borang soal selidik pada hari pemeriksaan antenatal untuk anda jawab. Anda dikehendaki menjawab setiap borang soal selidik pada ketika itu iaitu pada hari pemeriksaan antenatal dan dikehendaki memulangkannya kepada pengkaji atau staf klinik sebelum meninggalkan klinik. Sepanjang kajian ini anda dikehendaki untuk terus mengambil suplemen zat besi dan vitamin lain yang dibekalkan oleh doktor anda.

Pihak pengkaji hanya akan mengambil keputusan darah anda daripada buku antenatal anda. Pengambilan darah untuk paras haemoglobin adalah sebahagian daripada rutin pemeriksaan antenatal anda. Setelah tamat kajian ini, wanita hamil daripada kumpulan kawalan juga akan diberikan maklumat anemia semasa kehamilan menggunakan modul MYPINKMOM ini.

***Research Background and Objectives***

*Anaemia in pregnancy can bad effects to mothers and babies. This research is aimed to study factors of anaemia in pregnancy and to educate pregnant women to improve this condition. There are 360 pregnant women from six antenatal clinics in Petaling district will join this research.* *As an experiment in this study, 180 pregnant women will be given information about anemia in pregnancy through WhatsApp using the MYPINKMOM module for 3 weeks. While 180 pregnant women will be given a pamphlet about anaemia during pregnancy.*

*You will be involved in this study for 8 weeks only. For those with information via WhatsApp, you will get information about anaemia during pregnancy in 2 sessions a week for 3 weeks and you will be required to write down when you have read the information given on the WhatsApp in a diary that also will be given to you. For those who get a pamphlet about anemia during pregnancy from a clinic staff, you are required to read it at home.*

*In the first week of study, a questionnaire will be given during the antenatal check-up day for you to answer. In the second, third and fourth week of study, information about anemia during pregnancy will be provided via WhatsApp or pamphlet. In the fifth week, you will be given a questionnaire on the day of the antenatal check-up for you to answer. In the sixth and seventh week, no information about anemia in pregnancy will be given. In the eighth week, you will be given a questionnaire on the antenatal examination day for you to answer. On that day, you will be given a set of questionnaires to be answered. Upon completion of the questionnaire, you are required to return it to the researcher or clinic staff before leaving the clinic.* *Throughout the study, you are required to take iron supplement and other vitamins prescribed by doctors daily.*

*The researcher will only trace your blood investigations result from your antenatal book. The blood taking for haemoglobin level is part of your antenatal care. Upon completion of the study, pregnant women from the control group will also be given information about anaemia in pregnancy using the MYPINKMOM module.*

**Instrumen Kajian/ *Study Instruments***

**Borang soal selidik**

Sebagai responden, anda akan diberikan borang soal selidik dan anda diminta untuk menjawab soalan-soalan dalam borang soal selidik tersebut dengan sebaik mungkin.

***Questionnaires***

*As the respondent, you will be given questionnaires forms and required to answer the questions as best as you can.*

**Kerahsiaan**

Semua maklumat peribadi yang diberikan dalam soal selidik tersebut adalah sulit. Tiada data pengenalan peserta kajian seperti nama pserta, nombor kad pengenalan dan nombor telefon akan diletakkan pada borang soal selidik. Setiap peseta kajian akan diberikan nombor koding. Hanya pengkaji sahaja dapat melihat rekod kesihatan peserta dan data kajian. Sebarang keputusan kajian yang akan digunakan akan dipersembahkan sebagai hasil kolektif, bukan dipersembahkan sebagai hasil individu. Semua data akan dirahsiakan dan digunakan untuk kajian ini sahaja.

***Confidentiality***

*All individual information given in questionnaires will be treated as confidential. There are no identification data, such as respondent’s name, ic number and phone number will be revealed in the questionnaire. Each respondent will be given a coding number. Only researcher can access medical record and research data. Any research findings to be used will be summarised as collective results, not as individuals. All data will be kept confidential and be used for this research only.*

**Hak sebagai responden**

Penyertaan anda sebagai reponden dalam kajian ini adalah sukarela. Anda mempunyai hak untuk menarik diri dari kajian ini pada bila-bila masa jika anda merasa tidak selesa di mana-mana peringkat penyelidikan dan tiada penalti akan diberikan. Jika penyertaan anda diberhentikan dalam kajian ini kerana sebab tertentu, penjagaan kesihatan antenatal akan diteruskan seperti biasa. Sebagai peserta kajian, anda tidak boleh melihat rekod kesihatan dan data kajian. Namun, anda boleh mengetahui keputusan kajian jika anda mahu. Anda sebagai peserta kajian akan dimaklumi sekiranya terdapat maklumat baru berkaitan persetujuan anda menyertai kajian ini.

***Respondent rights***

*This research requires voluntary participations from all respondents. You have the right to withdraw from this study at any time if you feel uncomfortable at any stage of the research and no penalties will be given. If your participation is stopped in this study for any reason, the antenatal care will be continued as usual**. As a respondent, you are not allowed to access your medical record and research data. However, you can be informed about this research findings if you wish to. You will be informed if there is new information relevant to consent of this study*

**Tindakan anda sebagai responden**

Anda hanya perlu menandatangani borang kebenaran bertulis untuk menunjukkan minat dan kesanggupan anda untuk mengambil bahagian dalam penyelidikan ini. Borang kebenaran bertulis ini mesti dikembalikan kepada penyelidik semasa kajian. Anda hanya perlu menjawab set borang soal selidik. Anda juga dikehendaki mengambil supplement zat besi dan vitamin lain setiap hari seperti yang diberikan oleh doktor. Anda juga perlu elak daripada mengambil ubat-ubatan yang dilarang oleh doktor anda. Pihak pengkaji hanya akan mengambil keputusan darah iaitu paras hemoglobin anda daripada buku pemeriksaan antenatal anda. Pengambilan darah adalah sebahagian daripada rutin pemeriksaan antenatal di klinik.

***Your action as respondent***

*You just need to sign in the written permission form to indicate your interest and willingness to participate in this research activity. This written consent form must* *be returned to the researcher during the study. You only need to answer a set of questionnaires. You are also required to take daily supplements of iron and vitamins as provided by your doctor. You should also avoid taking medicines that your doctor prohibits. Researcher will only trace your blood hemoglobin levels from your antenatal examination book. Blood taking for haemoglobin level is part of the routine antenatal examination at the clinic and is taken by trained clinic staff.*

***Faedah kajian***

Dengan menyertai kajian ini anda akan mendapat maklumat mengenai anemia semasa hamil. Dengan maklumat ini, anda dapat memperbaiki ilmu pengetahuan mengenai anemia semasa hamil, pengambilan suplemen zat besi yang betul dan pemakanan yang betul yang akan membantu meningkatkan paras hemoglobin anda. Dengan pelaksanaan kajian ini, modul MYPINKMOM boleh digunakan oleh kakitangan kesihatan ke atas wanita hamil dengan anemia semasa hamil dan ini akan menurunkan kes anemia semasa hamil pada masa akan datang.

***Benefits of the study***

*By participating in this study, you will gain information about anemia during pregnancy. With this information, you can improve the knowledge of anemia during pregnancy, the correct intake of iron supplements and proper nutrition that will help to increase your hemoglobin level. By implementation of this study, MYPINKMOM educational module can be used by medical staff on pregnant women with anemia in pregnancy and the cases of the anaemia in pregnancy can be reducedin the future.*

Risiko atau ketidakselesaan dalam kajian ini

Penyelidik akan mengesan tahap hemoglobin dari buku antenatal. Pengambilan darah dilakukan oleh kakitangan terlatih di klinik. Pengambilan darah untuk tahap haemoglobin ini adalah sebahagian daripada rutin prosedur pemeriksaan antenatal iaitu setiap 2 minggu atau setiap bulan. Pengambilan darah boleh menyebabkan sakit dan lebam di tempat pengambilan darah. Walaubagaimanapun, intervensi pengajaran dalam kajian ini adalah berisiko minima.

***Foreseeable risks or inconveniences of the study***

*In this study, the researcher will trace haemoglobin level from the antenatal book. Blood taking is done by trained staff in the clinic. It is part of the routine antenatal check-up done every 2 weeks or monthly. Blood taking may cause pain and bruises at blood taking site. Yet, the educational intervention in this study is associated with minimal risk.*

**Bayaran imbuhan atau bayaran balik**

Tiada bayaran imbuhan atau bayaran balik akan diberikan kepada peserta dalam kajian ini.

***Payment or reimbursement***

*There will be no payment or reimbursement will be given to the respondent*

Sebarang soalan bolehlah dikemukakan kepada saya jika anda mempunyai sebarang kemusykilan berkaitan kajian ini.

*Please do not hesitate to ask questions to the researcher should you need further clarifications on this study.*

Dr. Raudah binti Abd Rahman

Pelajar Doktor Kesihatan Awam (DrPH)/ Doctor of Public Health candidate

Pusat Perubatan Universiti Kebangsaan Malaysia (PPUKM)/ Universiti Kebangsaan Malaysia Medical Centre (UKMMC)

Tel: 019-6283088

Sekiranya anda mempunyai kemusykilan tentang hak-hak sebagai peserta kajian, sila hubungi *Malaysian Research Ethics Committee (MREC).*

*If you have any inquiry on rights as research subjects, please contact Malaysian Research Ethics Committee (MREC).*

MEDICAL RESEARCH AND ETHICS COMMITTEE (MREC)

Secretariat of National Institutes of Health (NIHSEC)

c/o Institute for Health Management

Jalan Rumah Sakit, Bangsar

59000 Kuala Lumpur

Telephone: +603 – 2282 9082 / 9085 / 4032 / 0491

Terima kasih atas kerjasama anda.

Thank you for your cooperation.

APPENDIX D: WRITTEN CONSENT FORM

I, ­­­_________________________________ ( IC Number: ______________________)

**AGREE / DO NOT AGREE** to participate in this research titled:

**Title: Development and Effectiveness of MYPINKMOM Educational Module for Pregnant Women with Anaemia in Petaling district**

1. I have read the Information for Respondent and understand the contents of the research and research purposes.
2. I understand that all information given here and all individual results from questionnaires are confidential which will be used for research purpose and for researcher’s reference only.
3. I also understand that all information obtained may be used for research publication but all personal details will not be disclosed at utmost confidential.
4. I understand that I have the right to withdraw my participation and permission at any time, whenever I feel uncomfortable during any stage of the research.

| __________________ | _________________ |
| --- | --- |
| (Respondent signature) | (Witness signature) |
| Name: | Name: |
| IC Number: | IC Number: |
| Date: | Date: |

BORANG KEBENARAN BERTULIS

Saya, _________________________________ (Nombor IC: ______________________) SETUJU / TIDAK BERSETUJU untuk mengambil bahagian dalam kajian ini yang bertajuk “Pembinaan dan Keberkesanan Modul Pendidikan Kesihatan MYPINKMOM bagi Wanita Hamil yang mengalami Anemia di daerah Petaling”.

1.Saya telah membaca Helaian Maklumat Subjek dan memahami kandungan penyelidikan dan tujuan penyelidikan.

2. Saya faham bahawa semua maklumat yang diberikan di sini dan semua hasil dari soal selidik adalah rahsia dan hanya akan digunakan untuk tujuan penyelidikan dan untuk rujukan penyelidik sahaja.

3.Saya juga memahami bahawa semua maklumat yang diperolehi boleh digunakan untuk penerbitan penyelidikan tetapi semua butiran peribadi subjek/responden kajian tidak akan didedahkan

4. Saya faham bahawa saya mempunyai hak untuk menarik balik penyertaan dalam kajian ini pada bila-bila masa sekiranya saya merasa tidak selesa pada mana-mana peringkat kajian

| (Tandatangan responden) |  | (Tandatangan saksi) |
| --- | --- | --- |
| Nama: |  | Nama: |
| Nombor IC: |  | Nombor IC: |
| Tarikh: |  | Tarikh: |
